# A Comprehensive Review of SLR Systems: Challenges, Datasets, and Unresolved Gaps

**DOI:** 10.3390/jimaging12080357

**Published:** 2026-08-05

**Authors:** Aigerim Yerimbetova, Ulmeken Berzhanova, Marek Milosz, Bakzhan Sakenov, Elmira Daiyrbayeva, Lyailya Cherikbayeva

**Affiliations:** 1 Institute of Information and Computational Technologies of the Committee of Science of the Ministry of Science and Higher Education of the Republic of Kazakhstan, Almaty 050010, Kazakhstan; aigerian8888@gmail.com (A.Y.); m.milosz@pollub.pl (M.M.); sbakzhan22@gmail.com (B.S.); cherikbayeva.lyailya@gmail.com (L.C.); 2 School of Engineering and Information Technology, META University, Almaty 050012, Kazakhstan; 3 Faculty of Information Technology and Artificial Intelligence, Farabi University, Almaty 050038, Kazakhstan; 4 Department of Computer Science, Faculty of Electrical Engineering and Computer Science, Lublin University of Technology, 20-618 Lublin, Poland; 5 Department of Software Engineering, Satbayev University, Almaty 050010, Kazakhstan

**Keywords:** sign language, sign language recognition, sensors, systems, methods

## Abstract

With the rapid advancement of sensor technologies, automated sign language recognition (SLR) has emerged as a critical enabler of inclusive communication systems for individuals with hearing and speech impairments. Although substantial research effort has been directed toward this domain, existing reviews lack a structured comparison of sensing modalities and do not systematically address the challenges of low-resource sign languages. This paper presents a comprehensive systematic review of sensor-based and multimodal SLR systems, covering 76 publications from 2021 to 2026 selected through a PRISMA 2020 protocol. We propose an original four-category taxonomy encompassing wearable sensor-based, contactless non-visual, vision-based, and multimodal systems, and provide a three-category methodological classification distinguishing conventional, machine learning, and deep learning approaches. The comparative analysis reveals that, despite notable progress, critical challenges persist: the absence of standardized datasets, limited cross-user generalization, insufficient multimodal fusion strategies, and inadequate representation of low-resource sign languages, including Kazakh Sign Language (KSL). The findings of this review establish a structured foundation for future research aimed at developing robust, scalable, and computationally efficient SLR systems.

## 1. Introduction

According to the World Health Organization, more than 5% of the world’s population, or approximately 430 million people, require rehabilitation due to hearing loss. By 2050, more than 700 million people, or one in ten, are projected to have significant hearing loss, highlighting the growing importance of communication support technologies for this population [1].

One of the most important, yet little-discussed, problems facing society is the limited capacity of the digital infrastructure to fully meet the needs of people with speech and hearing impairments. People with speech and hearing impairments remain one of the most socially vulnerable groups.

This issue is particularly relevant for Kazakhstan, where more than 30,000 people have hearing impairments, but it also reflects a broader international problem, especially for low-resource sign languages that remain insufficiently supported by modern technologies [2]. People with hearing impairments use sign language to communicate, which is not always understood by their hearing interlocutors. Mastering sign language, just like verbal language, is essential for the development of a deaf person’s personality, mental development, and social adaptation.

**Figure 1:**
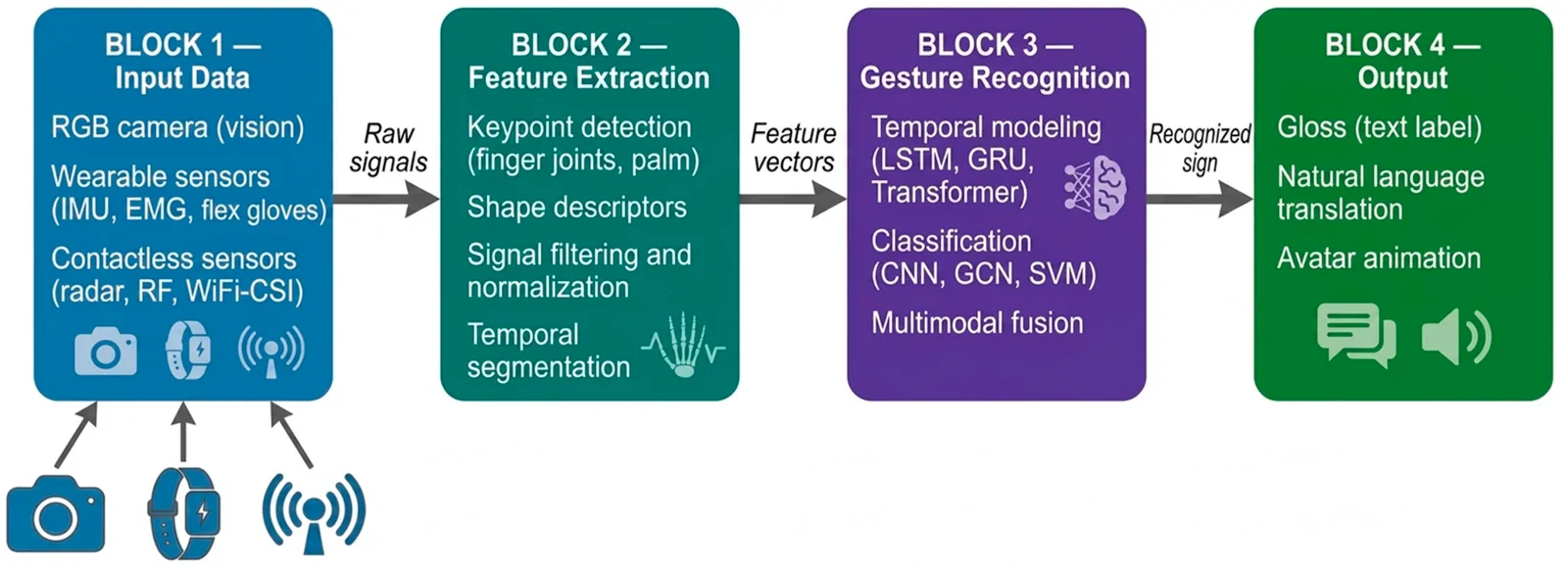
General architecture of an SLR system.

Input data are acquired through vision-based, wearable sensor-based, or contactless non-visual sensing modalities. Feature extraction produces structured representations that are passed to a gesture recognition module employing deep learning or conventional classification methods. The output layer generates a textual gloss, natural language translation, or avatar-based visualization (see Figure 1).

Sign language is a means of communication for people with hearing impairments, conveying information through hand, body, and facial movements. Sign language is a natural language, and its analysis utilizes methods developed in the study of spoken (auditory) languages, as it shares fundamental properties with them [3,4,5,6,7,8,9,10,11,12,13,14,15,16,17,18,19,20,21,22,23,24,25].

Table 1 presents the position of the current review in relation to two closely related survey articles by Amin et al. [4] and Najib et al. [7].

The survey by Amin et al. [4] provides a comparative overview of flex-resistive, vision-based, and hybrid sensor techniques for SLR; however, it does not apply systematic literature selection criteria, does not differentiate sensor modalities into a structured taxonomy, and does not focus on low-resource sign languages or bias in dataset construction. The review by Najib et al. [7] has supplied a wider variety of SLR methods through their review; however, they did not use a PRISMA-based selection protocol, nor did they provide any dedicated analysis of various types of wearables, contactless and multimodal sensing systems, or discuss any challenges related to low-resource languages. In contrast, this comprehensive review uses a PRISMA 2020 methodology on 76 publications from 2021 through 2026, presents an original four-category taxonomy for SLR systems (wearable sensor-based, contactless non-visual, vision-based, and multimodal), and proposes a three-category methodological classification to distinguish between conventional methods, machine learning, and deep learning techniques. Finally, this review emphasizes low-resource sign languages and incorporates an integrative research roadmap together with a qualitative discussion of potential dataset- and methodology-related biases. No formal study-level risk-of-bias assessment was performed.

Conceptually, the novelty of this taxonomy lies not merely in its four categories per se, but in explicitly crossing two orthogonal analytical dimensions—sensing modality (wearable, vision-based, contactless non-visual, multimodal) and methodological generation (conventional, machine learning, deep learning)—into a single joint framework. This allows, for example, a wearable-sensor deep learning system to be directly compared against a vision-based deep learning system on equal methodological footing, a comparison that single-axis taxonomies used in prior SLR reviews (which classify either by modality or by method, but not both jointly) cannot support.

The main contributions of the proposed work are as follows:

1. A systematic literature review was performed on 76 SLR studies published between 2021 and 2026 using a PRISMA 2020-guided selection protocol to provide a transparent, structured, and reproducible synthesis of the field.
2. A new structured taxonomy of SLR systems is proposed, consisting of four categories: (a) wearable sensor-based systems, (b) contactless non-visual systems, (c) vision-based RGB systems, and (d) multimodal systems; this taxonomy enables structured comparison across architectures.
3. Three categories of methodological approaches to sign language recognition are proposed: (a) conventional technique-based systems, (b) machine learning-based systems, and (c) deep learning-based systems; representative architectures for each category are fully covered.
4. Unresolved issues, such as the lack of datasets, static multimodal fusion strategies, limitations of temporal modeling, and low scalability, are identified and systematically discussed, and promising research plans in this area are presented.
5. A detailed analysis focusing on under-resourced sign languages, particularly KSL, is conducted, revealing important data challenges and highlighting the need for standardized, large-scale multimodal data collections for underrepresented sign language communities.

## 2. Materials and Methods

This review uses a systematic and structured approach to provide comprehensive and objective analysis of sign language recognition (SLR) systems. Leading databases, including Scopus, Web of Science, IEEE Xplore, and Google Scholar, were used to search and select scientific publications.

### 2.1. Literature Search and Study Selection

The literature search was conducted using keywords such as sensor-based sign language recognition, wearable sensors, Inertial Measurement Units (IMUs), contactless sensing, gesture recognition, and multimodal SLR. Particular attention was paid to recent and highly ranked publications reflecting the current state of research in this field.

The study selection process included several stages. Initially, duplicate records and irrelevant studies were excluded based on title and abstract screening. A full-text review was conducted according to the following predefined inclusion criteria: (i) direct relevance to wearable sensor-based, contactless non-visual, vision-based, or multimodal SLR systems; (ii) clear specification of the acquisition modality and dataset; and (iii) availability of sufficient methodological and experimental details, including at least one quantitative performance metric.

The exclusion criteria included duplicate records, studies not directly related to sign language recognition, papers lacking sufficient technical or experimental information, non-English publications, and studies outside the selected publication period.

Each selected publication was systematically analyzed and categorized according to sensor type, data collection methodology, employed models, and application domain. This process resulted in the proposed taxonomy, enabling a structured comparison of existing techniques and the identification of current challenges, research gaps, and future research directions.

The proposed review methodology was selected because it enables a consistent and objective comparison of heterogeneous sign language recognition systems developed using both visual and hardware-based sensing technologies. Unlike conventional narrative reviews, the PRISMA-guided systematic approach minimizes selection bias while ensuring transparent study identification, screening, and inclusion. Furthermore, categorizing the selected studies according to sensing modality, learning methodology, datasets, and application scenarios provides a unified analytical framework for comparing fundamentally different acquisition technologies, including camera-based systems, wearable sensors, contactless sensing, and multimodal approaches. This structured methodology therefore facilitates the identification of common research trends, technological limitations, and future research directions across diverse SLR paradigms.

**Figure 2:**
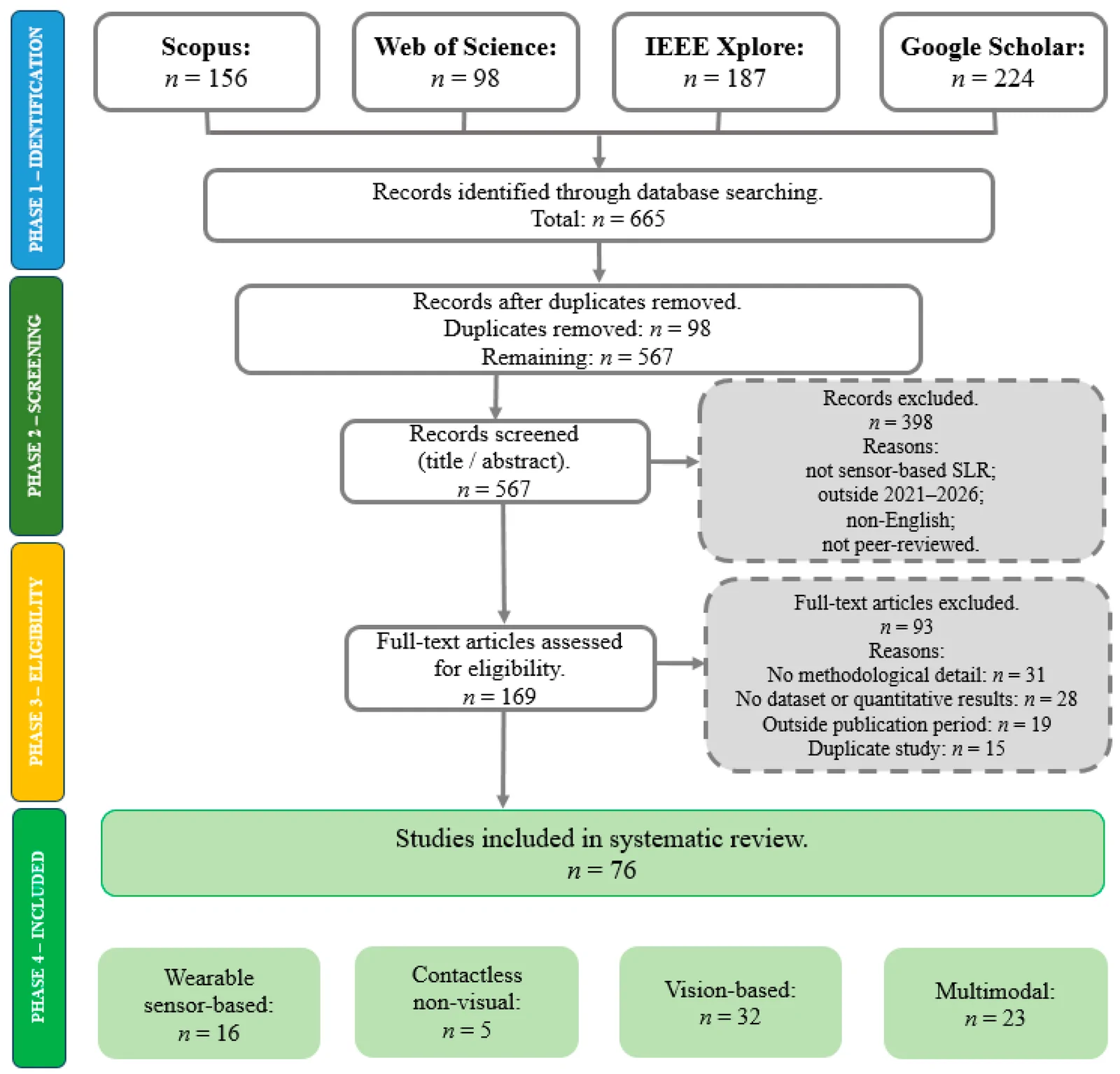
PRISMA 2020 flow diagram of the study selection process.

Through searches of the four information sources, a total of 665 records were identified. After removing 98 duplicate records, 567 unique records remained for title and abstract screening. At this stage, 398 records were excluded because they were outside the predefined SLR scope, outside the 2021–2026 publication period, published in a language other than English, not peer-reviewed, or otherwise not relevant to the review objectives. Consequently, 169 reports proceeded to full-text eligibility assessment. Of these, 93 reports were excluded because of insufficient methodological detail (*n* = 31), absence of dataset or quantitative performance information (*n* = 28), publication outside the predefined period identified during full-text assessment (*n* = 19), or duplicate reports identified during detailed full-text assessment (*n* = 15). Consequently, 76 original studies were included in the final systematic review (Figure 2). The search strategy combined terms describing the target task with terms representing the four sensing categories covered in this review: wearable sensor-based, contactless non-visual, vision-based, and multimodal systems. The following general Boolean expression was used and adapted to the syntax of each database: (“sign language recognition” OR “sign language gesture recognition” OR “isolated sign language recognition” OR “continuous sign language recognition” OR “sign language translation”) AND ((“wearable sensor” OR “smart glove” OR “data glove*” OR “flex sensor*” OR “IMU” OR “inertial measurement unit*” OR “accelerometer*” OR “gyroscope*” OR “magnetometer*” OR “sEMG” OR “electromyograph*”) OR (“vision-based” OR “computer vision” OR “camera-based” OR “video-based” OR “RGB” OR “RGB-D” OR “depth camera*” OR “skeleton-based” OR “keypoint*” OR “pose estimation” OR “hand tracking”) OR (“contactless sensing” OR “radar” OR “radio frequency” OR “RF sensing” OR “Wi-Fi CSI” OR “ultrasonic sensing” OR “acoustic sensing”) OR (“multimodal” OR “multi-modal” OR “sensor fusion” OR “cross-modal”)).

The search was applied to Scopus, Web of Science, IEEE Xplore, and Google Scholar. The general search expression was adapted to the syntax and search-field requirements of Scopus, Web of Science, IEEE Xplore, and Google Scholar. The search was restricted to English-language publications from 2021 to 2026 and was conducted between February and March 2026. The retrieval counts for each information source are reported in the PRISMA flow diagram. All co-authors participated in title/abstract and full-text screening, with the initial record pool divided approximately evenly among the author team and borderline cases resolved through joint discussion; a formal inter-rater agreement statistic was not computed, as this was not a fully duplicated independent screening process. Beyond the inclusion criteria stated above, studies were additionally required to report at least one quantitative performance metric, clearly specify the sensing modality and dataset used, and be published in a peer-reviewed journal or conference proceedings in English.

No formal study-level risk-of-bias scoring tool was applied in this review because the included studies were highly heterogeneous in terms of sensing modality, dataset design, recognition task, and evaluation protocol. Instead, methodological eligibility was assessed using predefined criteria, including peer-reviewed publication status, sufficient methodological description, explicit identification of the sensing modality and dataset, and reporting of at least one quantitative performance metric. Potential sources of bias, such as limited signer diversity, signer-dependent evaluation, small sample sizes, restricted recording conditions, and inconsistent evaluation protocols, were considered qualitatively in the comparative analysis and discussion. Accordingly, methodological eligibility assessment should not be interpreted as a formal risk-of-bias assessment.

A completed PRISMA 2020 checklist, cross-referencing each checklist item to its corresponding location in this manuscript, is provided in Appendix C.

### 2.2. Analytical Framework and Taxonomy Development

To enable a systematic and consistent comparison of the studies included in this review, an analytical framework was developed based on two complementary dimensions: the sensing modality used for sign language data acquisition and the methodological approach employed for recognition. During the data extraction stage, each included study was examined with respect to its sensing technology, input data type, recognition methodology, dataset characteristics, and reported experimental outcomes.

Based on the primary sensing modality, the reviewed SLR systems were organized into four categories: (i) wearable sensor-based systems, including approaches based on inertial measurement units (IMUs), flex sensors, electromyography (sEMG), smart gloves, and related wearable devices; (ii) contactless non-visual sensing systems, including radar, radio-frequency (RF), Wi-Fi channel state information (Wi-Fi CSI), and related sensing technologies; (iii) vision-based systems, primarily relying on RGB, RGB-D, depth, skeleton, or keypoint-based representations; and (iv) multimodal systems, which combine two or more complementary sensing modalities.

In parallel, the included systems were classified according to their methodological approach into three broad groups: (i) conventional or rule-based techniques, including template matching and traditional signal-processing methods; (ii) machine-learning-based approaches, such as Support Vector Machines, k-Nearest Neighbors, Random Forests, and related classifiers; and (iii) deep-learning-based approaches, including convolutional neural networks, recurrent neural networks, LSTM/GRU architectures, graph-based models, Transformers, and multimodal deep-learning frameworks.

These two dimensions were considered jointly rather than independently. Thus, for example, a wearable sensor-based deep-learning system can be compared with a vision-based deep-learning system while preserving the distinction between their sensing modalities. This analytical structure was subsequently used to organize the studies presented in the Section 3, support cross-category comparisons, and identify common technological limitations and research gaps across heterogeneous SLR paradigms.

**Figure 3:**
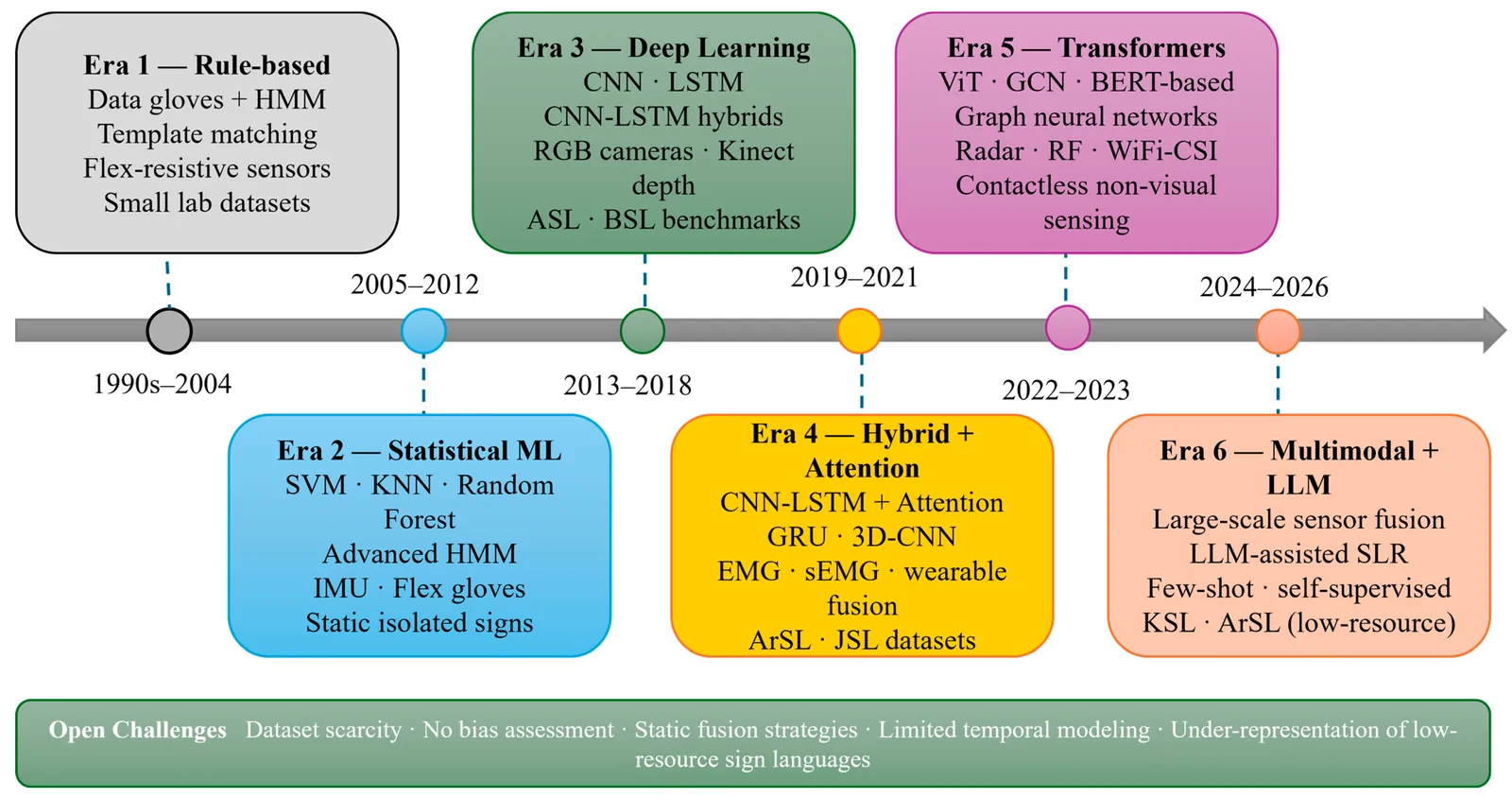
Evolutionary trajectory of sign language recognition systems from rule-based methods (1990s) to multimodal deep learning and LLM integration (2024–2026). Block color intensity reflects methodological complexity. A summary of the identified open-research challenges from this present review can be found in the section of the document titled Summary of Open Challenges.

Figure 3 provides a complementary overview of the broader methodological evolution of SLR systems, illustrating the transition from early rule-based and statistical approaches toward deep-learning, Transformer-based, multimodal, and large-language-model-assisted systems.

## 3. Results: Review of Sensor-Based and Multimodal SLR Systems

### 3.1. Wearable Sensor-Based Systems

Recently, there have been great improvements in sensor-based and wearable SLR systems. Sensor-based and wearable systems capture motion of the upper extremity, and also capture the inertial information and the electrical activity of muscles, in order to provide real-time gesture detection for individuals with hearing and speech limitations. Sensor-based and wearable SLR systems use various combinations of sensors, such as Flex Sensors, inertial sensors (accelerometers, gyroscopes, magnetometers), Electromyography (EMG), and other sensors, to collect data regarding the movement of the fingers and hands.

**Figure 4:**
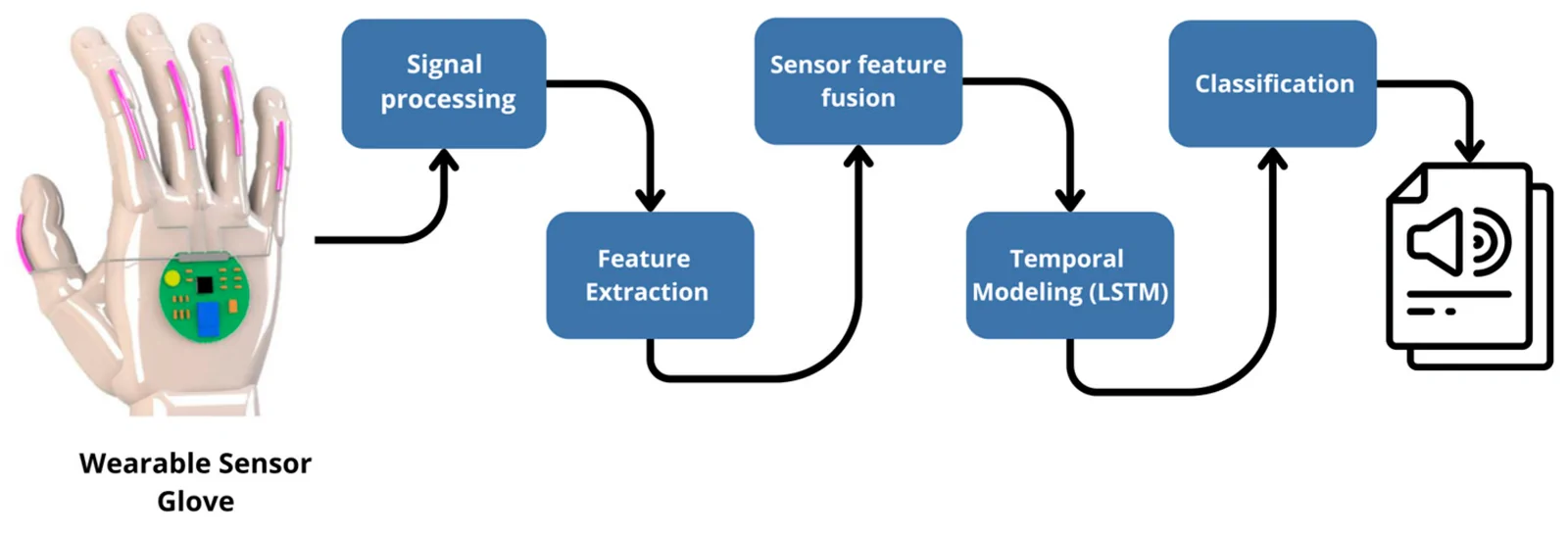
Sensor-based SLR system architecture.

A basic layout of the sensor-based SLR system structure is shown below in Figure 4. The architecture of a gesture recognizer using sensor data has six components:

- Sensors: This stage involves gathering data through finger sensors that identify a person’s hand movements through an inductive sensor located on the rear of the hand that processes and transmits the information.
- Signal preprocessing: This step includes removing noise from data and filtering and normalizing the sensor data.
- Feature extraction: It captures the significant characteristics of a gesture, including amplitude, velocity, and tilt angle differences, which help differentiate among various gestures.
- Sensor feature fusion: This step aggregates the feature streams extracted from the wearable sensor’s multiple channels (e.g., inductive/flex sensor signals) into a single combined feature representation before temporal modeling is applied.
- Temporal modeling: This step uses temporal processing models to analyze the collected data’s sequences by considering when each movement occurs to create a record of the trajectory and position of the hand throughout the entire video stream.
- Classification: Final processing within the neural network assigns a category to the collected data.
- Output: The recognized gesture may be converted to either text or synthesized speech.

In the 2021 study by Na et al. [26], accelerometer measurements were used to classify Korean Sign Language alphabet gestures. Let $a_{t}\;=\;{\left[a_{x}(t),\;a_{y}(t),\;a_{z}(t)\right]}^{T}$ denote the three-axis accelerometer readings at time t. The feature vector is extracted from the accelerometer sequence using statistical descriptors:

(1) $$f\;=\;[\mu_{x},\;\sigma_{x},\;\mu_{y},\;\sigma_{y},\;\mu_{z},\;\sigma_{z}],$$

where μ and σ represent the mean and standard deviation of each axis over the gesture duration.

The classification is performed using a Support Vector Machine (SVM). The decision function is defined as:

(2) $$f(x)\;=\;\mathrm{sign}\left(\sum_{i=1}^{n}\alpha_{i}y_{i}K(x_{i},x)\;+\;b\right),$$

where $\alpha_{i}$ are Lagrange multipliers, $y_{i}$ are training labels, K(.,.) is a kernel function, and b is the bias term.

The system captures motion information using a single three-axis accelerometer. Temporal accelerometer signals are transformed into a fixed-dimensional feature vector using statistical descriptors computed over the duration of a gesture. The Support Vector Machine constructs a separating hyperplane in a high-dimensional feature space. To classify alphabet signs accurately, an SVM classifies signs using maximal margin between gesture classes. This maximizes robustness and reduces the amount of hardware required (only one inertial sensor) by using known existing classification methods based on kernels.

While an accelerometer provides a possible solution for reduced hardware and demonstrates that it is feasible to perform alphabet classification with accelerometers, there are still significant limitations with the use of an accelerometer-based approach. First, because it relies solely on accelerometer signals, the ability to accurately capture the detailed fine motor motion of the fingers is limited; this is especially true when attempting to differentiate between similar signs. Second, once temporal signals are aggregated into statistical data, the fine sequential movement of the finger motion trajectories and transitions is no longer available to the model. Thus, a motion model developed from accelerometers alone does not leverage the temporal dependencies of motion and will become problematic as the vocabulary of mappings of signs continues to grow larger. Sensor-only motion models derived from accelerometers will also exhibit great variability due to differences in speed of execution and battery life, as well as different styles of individually assigned motion, which will have a negative impact on the ability to generalise across different signers. This suggests that, for robust SLR, the incorporation of multi-modal sensor technology, as well as sequence-aware learning models, would be particularly beneficial.

In the 2021 research by Rinalduzzi et al. [27], a magnetic positioning system with wearable transmitter nodes is used to measure 3D finger position and orientation, the data from which is processed by a machine learning algorithm to recognize static alphabet gestures with an accuracy of about 97%.

Subsequently, in the 2021 study by Wen et al. [28], a wearable sensor glove is used to capture hand and finger movements to recognize gestures and sign language sentences, which are then processed by a deep learning model to translate into text and speech.

A smart glove with an auxiliary thread and an Artificial Neural Network (ANN) showed an accuracy of 98.5% for 26 letters of the English sign language alphabet [29].

In the 2022 study by Zhou et al. [30], the authors combine smartwatch-based inertial sensing with LSTM networks to achieve continuous feature recognition.

Let $s_{t}\in R^{d}$ denote the multi-feature sensor vector extracted at time t including accelerometer and gyroscope features.

The BLSTM computes forward and backward hidden states:

(3) $${\vec{h}}_{t}\;=\;{\mathrm{LSTM}}_{f}(s_{t},\;{\vec{h}}_{t-1}),$$

(4) $${\overset{\leftarrow }{h}}_{t}\;=\;{\mathrm{LSTM}}_{b}(s_{t},\;{\overset{\leftarrow }{h}}_{t+1}),$$

and the combined hidden representation is:

(5) $$h_{t}\;=\;[{\vec{h}}_{t}\|{\overset{\leftarrow }{h}}_{t}],$$

The final predicted gesture sequence is obtained through sequence decoding:

(6) $$\hat{Y}\;=\arg\;\underset{Y}{\max}P(Y|X),$$

The system leverages inertial data from smartwatches worn on the wrist. The multi-feature extraction converts raw data from the sensors into a composite representation of motion dynamics. The Bidirectional Long Short-Term Memory (BLSTM) networks allow for training the sequence data both forward and reverse so that the model can take advantage of all past and future context when determining the next gesture element to be recognized. This is particularly important for accurate recognition of continuous sign sequences as opposed to using only unidirectional recurrent models. The softmax layer produces the class probabilities at each time step being processed, and the sequence decoding produces the final recognized gesture sequence.

The BLSTM multi-feature framework provides an improved representation of temporal modeling through the use of bidirectional context; however, there are still several limitations. The system primarily relies on inertial sensing and does not utilize complementary modalities such as EMG or visual input, which might limit their ability to discriminate fine-grained articulation differences. While BLSTM networks can capture contextual dependencies, they may not be effective in capturing long-range temporal dependencies, nor can they effectively model complex grammatical structures in extended continuous signing. Furthermore, feature extraction remains partially manual and not fully trained, which may limit how well the model adapts across different datasets and users. Also, real-world deployment issues such as asynchronous sensor sampling, signal noise, and variability across different users are not fully addressed. These factors suggest that transformer-based architectures, adaptive multimodal fusion mechanisms, and domain adaptation strategies could further improve continuous smartwatch-based SLR systems.

Static American Sign Language (ASL) poses are performed using MLP and low-cost solutions with flexible sensors and IMUs that enable autonomous recognition without video cameras [31].

In the 2023 study by Kalandar and Dworakowski [32], a low-cost minimalist sensor system utilizing three Flex sensors has been proposed for the purpose of recognizing dynamic ASL words. The approach is based on the analysis of temporal sequences of gestures using machine learning algorithms (Random Forest, SVM, KNN), achieving an accuracy of up to 99%. Despite the limited number of sensors, the system demonstrates high efficiency, indicating potential for the development of energy-efficient and wearable solutions for sign language recognition.

In the work of researchers [33], the IRDC-Net architecture used a convolutional neural network with residual convolutional modules and augmented convolutional modules to process multi-channel surface electromyography (sEMG) signals.

Let $e_{t}\in R^{M}$ denote the multichannel sEMG signal at time t, where M represents the number of electrodes.

The dilated convolution operation is defined as:

(7) $$(F_{d}*W)(t)\;=\;\sum_{k}F(t\;-\;d\cdot k)W\left(k\right),$$

where d is the dilation rate, allowing enlarged receptive fields without increasing parameter count.

Residual connections are incorporated as:

(8) $$F^{\left(l\right)}\;=\;H\left(F^{\left(l-1\right)}\right)\;+\;F^{\left(l-1\right)},$$

where H(⋅) represents the residual mapping.

The system utilizes multichannel sEMG signals that capture muscle activation patterns associated with hand gestures. Unlike inertial sensors, sEMG measures muscle activity to obtain a direct measurement of muscle activity, allowing for the identification of subtle articulation patterns. The IRDC-Net architecture uses convolutional layers to extract the temporal and spatial patterns found in an sEMG sequence. Additionally, dilated convolutions increase the size of the receptive field so the network can learn to capture long-range dependencies between input features with minimal increase in computational costs. Further, residual connections stabilize training of deep networks and aid in gradient flow. Therefore, the network can automatically learn to extract discriminative features (patterns) from the input sEMG signal via hierarchical feature learning, since it learns to identify the features of interest from the raw sEMG signals, rather than requiring that handcrafted features be created. Finally, the output of the last softmax layer produces posterior probabilities associated with gesture classes.

While the IRDC-Net architecture has demonstrated high accuracy on sEMG signals obtained via multiple electrodes for many gestures, there are still many challenges that need to be addressed before it is widely applicable. EMG measurements are sensitive to electrode misalignment, muscle fatigue, and skin-to-electrode contact resistance variations, which can significantly affect signal stability across sessions and users. Therefore, generalizing the model across user-to-user and session-to-session scenarios remains difficult. In addition, the architecture focuses only on muscle activity patterns, without incorporating additional kinematic information from inertial sensors, such as hand orientation and movement trajectories. Such reliance on a single modality may reduce robustness when muscle signals alone are insufficient to distinguish semantically similar movements.

Even though dilated convolutional layers increase the receptive field, there are no explicit temporal sequence modeling mechanisms (recurrent layers, attention-based encoders) built into the network. Therefore, long-range dependencies in terms of time, and the dynamics between gestures, may not be optimally captured. Finally, deep convolutional models require large amounts of annotated training data in order to obtain stable models for generalization. However, current publicly available datasets for sign language based on sEMG are small, leading to a higher risk of overfitting, as well as fewer opportunities to scale up with larger vocabularies. This creates a need for multimodal integration, domain adaptive techniques, and temporally aware learning frameworks to improve the robustness and generalization ability of sEMG-based wearable SLR systems.

Android systems with IoT gloves and mobile apps support multiple sign languages [34].

A wearable inertial motion capture system using 25 IMUs and CNN + Bi-LSTM has demonstrated high accuracy in sentence recognition and translation [35], and the system in this study [36] has demonstrated high accuracy in sign language translation tasks.

A series of motion sensors and reduced-flexibility sensors were used in the work [37] to recognize alphabetic gestures of static hand shapes. Let $x\in R^{d}$ denote the feature vector composed of sensor measurements is defined as:

(9) $$f\;=\;[f_{1},\ldots ,f_{k},\;a_{x},\;a_{y},\;a_{z}],$$

where $f_{1},\ldots ,f_{k}$ represent flex-sensor values and $a_{x},\;a_{y},\;a_{z}$ denote accelerometer components.

The glove uses a minimal number of sensors to reduce hardware complexity while maintaining recognition capability. Each static finger posture is represented as a fixed-dimensional feature vector containing flex and inertial information. The classification step applies to a probabilistic discriminative model (e.g., logistic regression or shallow neural network) to estimate class membership. The predicted gesture corresponds to the class with maximum posterior probability. The goal of this system is to provide a balance between recognition accuracy, with fewer sensors, a less complex, cost-efficient piece of hardware, and comfort for the user.

The glove with fewer sensors has shown that sufficient accuracy can be achieved with limited hardware; however, there are still many limitations. This is due to the fewer sensors; therefore, the data collected will be less extensive about the captured motion of the fingers, which may affect whether or not the system can distinguish between fingers that are in very similar configurations. This system has been built mainly to recognize static signs when fingers are held still, and the design does not incorporate and model the motions of the fingers over time in order to perform Continuous Sign Language Recognition (CSLR). The classification approach used in this system uses a fixed-length representation of the features collected, which does not take into account how the motions of the fingers occurred; therefore, it does not incorporate a sequential relationship nor the biomechanical constraints associated with how people naturally move their fingers. Lastly, with reduced coverage of sensors around the fingers, the system is more likely to be affected by measurement error, calibration drift, and individual differences in the shape of users’ hands when using the system. Overall, these challenges highlight the need for future work to explore learning methods that can adapt to the user’s needs and create a system that can model the motions of the fingers over time to provide more accuracy and provide a larger full-body scale of ASL in a wearable system for users.

In the 2025 study by Kader M.A. et al. [38], a machine learning classifier based on sensor features is used. The feature vector obtained from the wearable sensor is used. The feature vector $x\in R^{d}$ obtained from the wearable sensor is used.

The output probability distribution across gesture classes is calculated using softmax:

(10) $$P(c|h)\frac{\exp\left(w_{c}^{T}\;h\right)}{\sum_{c=1}^{C}\exp\left(w_{k}^{T}\;h\right)},$$

where h is the feature vector, $w_{c}$ is the learnable weight vector for class c, and C denotes the number of gesture classes.

The predicted gesture class is determined by:

(11) $$\hat{y}\;=\;\arg\underset{c\in \{1,\ldots ,C\}}{\max}P(c|h),$$

where $\hat{y}$ is the predicted class label.

The system processes features captured from wearable sensors through a Multi-Layer Perceptron (MLP) classifier. The data input into the system consists of a static or combined motion feature vector as the input. A nonlinear transformation is performed in the hidden layer of the classifier for the purpose of learning the discriminative representations of the features captured by the sensors. The final classification of an input feature will be made according to the greatest posterior distribution (probability) among all the predefined gesture classes provided by the softmax layer. This system is capable of classifying gestures with a moderate level of computational complexity in a relatively quick amount of time, making it appropriate to use in a real-time communication system.

Despite its practical use, several limitations remain:

1. Lack of Temporal Depiction. The proposal is built upon the idea that the feature vector x is constant throughout all dynamic gestures and therefore does not include any type of temporal relationship among the sequential motion within dynamic gestures; however, sign language incorporates many types of sequential motions and should be modeled using some form of recurrent or sequential modeling techniques.
2. Limited Feature Representation Power. MLPs are limited in their ability to capture complex non-linear articulation dynamics due to their shallow architecture. More complex architectures, like CNNs, LSTMs, or Transformers, would be better suited for learning hierarchical abstractions of motion.
3. Sensitivity to Feature Engineering. Performance is reliant on the ability to manually design features for extraction. The limitation of not having an end-to-end learning approach limits the ability to adapt to new datasets and sensor configurations.
4. Scalability Constraints. Thus, as the number of gesture classes grows, it is likely that linear classification will not be able to continue to keep discriminative boundaries from one class to another without developing a much deeper representation of the data.
5. Limited Generalization Across Users. Model robustness could be lessened by user-dependent movement style variation and placement of sensors, particularly due to lack of normalization and no method to adapt to different domains. Based on these limitations, it is recommended that additional sequence modelling, training of feature representations, and generalized adaptation strategies be implemented in future wearable SLR systems to increase robustness and scalability.
6. The review’s screening process did not include a formal inter-rater agreement statistic, as literature screening was divided among co-authors rather than conducted as a fully duplicated independent process by each reviewer.

Nepali sign language is recognized using Hall sensors and an IMU via an MLP on an embedded controller [39].

The study in ref. [40] presents a wearable multi-channel tactile sensor based on smart textile materials (triboelectric threads in gloves and sleeves) that records deformations and contact movements of fingers and wrists to recognize gestures with an accuracy of 94.66%.

In the 2025 study by Hazri et al. [41], a sensor glove with five flex sensors installed on the fingers to register bends is proposed, providing recognition of fingerprint gestures in real time using the Arduino MEGA 2560 platform.

In general, sensor systems utilize algorithms for real-time performance, mobility, versatility, and energy efficiency. Innovations include minimalist sensor design, integration with VR/AR, and mobile apps. Key limitations include static/dynamic gesture recognition, sensor noise, individual user differences, and hardware limitations. Along with the development of wearable communication devices, significant progress has been made in non-invasive, non-visual surveillance methods.

### 3.2. Contactless Non-Visual Sensing Systems

Contactless, non-visual recognition methods represent a promising alternative to wearable devices, eliminating issues related to hygiene, individual anthropometry, and dependence on lighting conditions.

The Radio Frequency-based Continuous Sign Recognition (RF-Csign) system for sign language recognition based on Radio Frequency Identification (RFID) signals is developed. To improve the expressiveness of features, a Large Kernel Convolution is applied, allowing for taking into account long-range dependencies in spatiotemporal data. To improve the robustness of the model, the Normalization-based Attention Module (NAM) is used, reducing the effect of overfitting and improving the generalization ability. Additionally, transfer learning is applied for cross-domain scenarios and new users. Experimental results show an overall accuracy of up to 99.17%, as well as 97.5% and 96.67% for new environments and users, respectively [42]. The RF-SL system is proposed as a contactless RFID-based sign language recognition system that does not require wearing tags. To improve data quality, static reflection noise removal is applied, as well as an improved Varri+ segmentation algorithm for gesture boundary detection. A hybrid Convolutional Neural Network–Bidirectional Long Short-Term Memory (CNN-BiLSTM) model, which combines spatial and temporal characteristics of signals, is used for feature extraction. The system demonstrates an average accuracy of 96.8%, and 96.3% for new users, showing robustness in various acoustic-radio frequency conditions (weak and strong multipaths) [43].

The UltrasonicGS system, a contactless method for gesture recognition based on ultrasonic signals, is proposed. To increase the training set, a Generative Adversarial Network (GAN) is used to create synthetic acoustic data. To process the sequences, a Convolutional Recurrent Neural Network (CRNN) architecture is used with the addition of a Connectionist Temporal Classification (CTC) layer, which allows for the alignment of input and output sequences for continuous gestures. Experiments show an accuracy of 98.8% for isolated gestures, as well as 92.4% and 86.3% for continuous and complex gesture sequences, respectively [44]. For energy-efficient gesture recognition, Dynamic Vision Sensor (DVS) is used in combination with Spiking Neural Networks (SNNs), which provide processing of spatiotemporal data. The paper also proposes specialized gesture datasets based on event data and a training method with spatiotemporal backpropagation. Experimental results show recognition accuracy of up to 77% [45].

Leap Motion is a unique system based on infrared (IR) sensors that can record 3D joint coordinates without analyzing the video stream. Using the Dynamic Time Warping (DTW) algorithm for time series matching, it achieved an accuracy of 95.17%, which ensures robustness to variations in the speed of execution of the gesture and independence from the visual background [46].

Thus, contactless gesture recognition systems use radio signals, ultrasound, and 3D sensors, along with deep and classical models, to ensure privacy, autonomy and environmental robustness. The main limitations include sensitivity to small movements, sensor noise, computational resources, and the need for precise equipment placement. The transition to RGB data opens up powerful deep learning tools that allow for efficient extraction of semantic information from video streams, making this approach a dominant approach for classifying large-scale sign language datasets.

### 3.3. Vision-Based Systems

Video-based sign language recognition systems use RGB video, depth cameras, and computer vision techniques to automatically extract spatial and temporal features of gestures. Based on video data, modern systems extract spatial and temporal features to analyze gesture dynamics [47,48,49,50]. Attention mechanisms and multi-feature attention are implemented in the architecture of deep learning models—between the feature extraction and temporal processing stages—which allows for the efficient selection of the most informative frames and movement trajectories, significantly increasing robustness to complex backgrounds, video stream redundancy, and individual differences between performers [51,52,53]. The use of data augmentation and transfer learning methods in systems such as Deep Learning-based Sign Language Recognition System (Deepsign) helps overcome the problem of the limitations of specialized datasets [54,55].

A significant portion of research is focused on the use of hand, face, and body keypoints extracted using MediaPipe Holistic or OpenPose Human Pose Estimation Framework (OpenPose) tools [56,57,58]. To model anatomical relationships, spatiotemporal graph networks such as Spatial Temporal Graph Convolutional Network with Long Short-Term Memory (STGCN-LSTM) [59] are used, which analyze the phonological characteristics of gestures, achieving an accuracy of 98.42% and robustness to visually similar movements. This approach allows for a transition from the analysis of full frames to a skeletal modality, which dramatically reduces the computational load and enables the systems to work on mobile devices and in web interfaces [60,61,62]. Spatial Temporal Graph Convolutional Network (ST-GCN)-like and graph neural network approaches, including Sign Language Graph-based Recognition Network (SIGNGRAPH) and hybrid GCN architectures, as well as multithreaded models based on graph convolutions, demonstrate robustness to occlusions and background changes by using a skeletal representation of gestures and modeling spatiotemporal dependencies of joints [63,64,65].

To process complex two-handed gestures and continuous signed speech, Transformer-based architectures with Multi-Head Self-Attention (MHSA) are used, which effectively handle long sequences despite the increased memory consumption [66,67]. To process complex sequences, Transformer-based and Gated Recurrent Unit (GRU)-based architectures with attention mechanisms are used. For example, a system for Korean sign language demonstrates recognition accuracies of 99.87% and 100%, respectively, while maintaining robustness under changing lighting conditions [68]. In this paper, a method for sign language recognition based on two video views is proposed. First, sequence alignment is performed, after which a model that extracts spatiotemporal features from both views is applied. To fuse the information, a Front-View Guided Early Fusion (FvGEF) strategy is used, allowing for efficient integration of data from different views. Experiments show a significant improvement in accuracy compared to single-view approaches, especially under occlusion. The main contribution is the use of multi-view information to improve the robustness of SLR [69].

The study in ref. [70] investigates the application of SNN in SLR. Compared to conventional neural networks, SNN is energy-efficient in terms of processing time. Experiments using the Word-Level American Sign Language Dataset 300 (WLASL300) show slightly better accuracy and a significantly reduced number of neural activations, making this approach promising for energy-efficient real-time systems. Innovative approaches such as the use of GANs have been aimed at addressing deep semantic and contextual dependencies in continuous sign language [71]. Also, alternative architectures such as the state-space model (SSM), which is implemented as part of the Mamba approach, have been able to effectively model long sequences and increase computational efficiency in feature extraction [72].

Although providing high accuracy, common limitations of RGB systems include sensitivity to lighting quality, limited vocabulary for dynamic gestures, and difficulties in fully protecting regions of interest [73,74]. A lightweight dynamic sign language recognition system based on a minimal angular geometric representation is proposed in [75]. Compact angular descriptors are used instead of a full set of keypoints, significantly reducing computational complexity. BiLSTM is used to model temporal dynamics. The method demonstrates high accuracy (approximately 99%) with low computational costs, making it suitable for mobile and real-time applications.

The authors demonstrated in their work [76] that it is based on a graph convolutional network (GCN) enhanced with multi-scale spatial modeling and attention mechanisms.

Let the human skeleton be represented as a graph $G\;=\;\left(V,\;E\right)$. V denotes the set of joints and E denotes anatomical connections.

Let $A\in R^{J\times J}$ denote the adjacency matrix of the graph, and $X_{t}\in R^{J\times F}$ denote joint features at time t, where J is the number of joints and F is the feature dimension (e.g., coordinates, velocity).

The spatial graph convolution is defined as:

(12) $$H_{t\;}^{\left(l+1\right)}\;=\;\;\sigma \;({\tilde{D}}^{-1/2}\tilde{A}{\tilde{D}}^{-1/2}H_{t}^{\left(l\right)}W^{\left(l\right)})\;,$$

where $\tilde{A}\;=\;A\;+\;I$ is the adjacency matrix with self-loops; $\tilde{D}$ is the degree matrix; $W^{\left(l\right)}$ is the learnable weight matrix; $\sigma \left(\cdot \right)$ is a nonlinear activation function.

Multi-scale spatial modeling is introduced by combining multiple adjacency matrices:

(13) $$H_{t\;}^{\left(l+1\right)}\;=\;\sum_{s=1}^{S}\sigma \;({{\tilde{D}}_{s}}^{-1/2}{\tilde{A}}_{s}{{\tilde{D}}_{s}}^{-1/2}H_{t}^{\left(l\right)}W_{s}^{\left(l\right)})\;,$$

where S represents different spatial scales.

The attention mechanism assigns adaptive weights:

(14) $$\alpha_{t}\;=\;\mathrm{softmax}\;\left(\phi \left(H_{t}\right)\right),$$

and the attended representation becomes:

(15) $${\tilde{H}}_{t}\;=\;\alpha_{t}⊙H_{t},$$

where ⊙ denotes element-wise multiplication.

This model represents the human body as a spatial graph, where joints are nodes and bones are edges. Graph convolution captures the structural articulation patterns between joints. Multi-scale modeling allows the network to learn local and global co-dependencies. The attention mechanism can further improve the recognition area by dynamically measuring spatial features. Temporal aggregation allows for dynamic sign language recognition by modeling the evolution of motion between frames. This hybrid design provides a single spatio-temporal framework that combines structural, multi-scale, and attention mechanisms.

Although attention-based GCNs improve the structural modeling of skeletal movements, some limitations remain. The graft topology is predefined and may not optimally represent the different linguistic dependencies and interactions in each sign language. Since the attention mechanism operates mainly at the spatial feature level, it may not be able to fully represent the temporal and semantic dependencies of long sign language sequences. Furthermore, skeleton-only modeling ignores non-manual cues such as facial expressions and subtle hand shapes, which are important in sign language grammar. The architecture also predicts reliable articulation detection, but shielding and sensory noise can reduce accuracy. Future research directions include adaptive graph learning, cross-modal integration with visual and wearable signals, and transformer-based temporal modeling to better represent hierarchical language structures.

A Sign Language Action Transformer Network (SLATN) is proposed for joint recognition of manual and non-manual gestures. The model uses a spatio-temporal transformer block to extract features from video sequences and an attention mechanism to focus on hands and faces. The system also includes gesture localization via bounding-box predictions. An accuracy of 82.66% is achieved on the Pakistan Sign Language Manual and Non-Manual Dataset (PkSLMNM) dataset with moderate computational complexity (94.13 GFLOPs) [77]. In the next proposed system, LSTM is used to process the motion features sequentially, and MediaPipe is used to extract keypoints. High accuracy with low latency is achieved [78].

RGB systems remain critically dependent on lighting conditions and direct visibility of the object. Therefore, modern research is shifting toward the development of hybrid and multimodal systems.

### 3.4. Multimodal Systems

Multimodal sign language recognition systems with heterogeneous data sources are approaches that combine multiple sensors and data modalities to improve accuracy, resilience to noise, hand occlusion, and gesture variability. The core principle of such systems is the combination of visual streams (RGB video), skeletal coordinates, optical flow, and wearable sensors, which compensates for the limitations of each individual modality.

#### 3.4.1. Vision-Based Multimodal Fusion Systems

A system for KSL is proposed that uses You Only Look Once Pose Estimation (YOLO-Pose) to extract skeletal coordinates and optical flow to analyze motion between frames. Features are combined via feature fusion and fed into BiLSTM for temporal sequence modeling. The model is robust to variations in gesture speed and operates in real time with high accuracy [79].

The proposed framework for dynamic SLR combines multimodal spatiotemporal features and 3D deformable convolution [80].

Let $X\in R^{T\times H\times W\times C}$ denote the input spatio-temporal tensor, where T is the number of frames, (H,W) are spatial dimensions, and C is the number of channels.

In deformable 3D convolution, learnable offsets $\Delta p_{n}$ are introduced:

(16) $$Y(p_{0})\;=\;\sum_{p_{n}\in R}W(p_{n})X(p_{0}\;+\;p_{n}\;+\;\Delta p_{n}),$$

where $\Delta p_{n}$ allows adaptive spatial–temporal sampling.

For multimodal fusion, let $F^{\left(k\right)}$ denote features extracted from modality k:

(17) $$F_{fusion}\;=\;\sum_{k=1}^{K}\alpha_{k}F^{\left(k\right)},$$

where $\alpha_{k}$ represents learned modality weights.

This model extends traditional 3D convolution by introducing deformable sampling offsets, allowing the network to adaptively focus on salient motion regions in both spatial and temporal dimensions. This is particularly useful for SLR, where hand articulation varies with speed, direction, and trajectory. Multimodality capabilities are combined with weighted aggregation, which allows the system to integrate additional spatiotemporal representations from different sensory streams. By combining warped convolution with multimodal synthesis, the architecture improves resilience to the variability of sign language and the complexity of dynamic gestures.

While deformation convolution and multimodal fusion provide flexibility, some limitations remain. Adaptive compensations increase the complexity and computational cost of the model, which prevents real-time deployment in sensor network environments. In addition, fusion mechanisms rely on static or globally learned weights rather than dynamic weights that are context-dependent over time. Deformation convolution improves spatiotemporal adaptation, but does not accurately model language-level dependencies or high-level semantic structures in continuous sign language. Also, training on such architectures requires large amounts of annotated data, which may not be readily available in resource-intensive sign languages.

A hybrid model combining a 3D-CNN for spatiotemporal feature extraction and a GCN for joint connectivity modeling is presented. An attention mechanism and multi-semantic fusion are additionally applied to combine features from video and skeletal data. The system demonstrates high accuracy and robustness to gesture variability [81].

A multi-stream recognition system for Turkish Sign Language is proposed, where RGB and optical flows are processed using Inflated 3D Convolutional Network (I3D), and pose data is processed using LSTM. Late fusion is then applied to combine the streams. The system demonstrates improved accuracy (up to 89.3%) compared to individual modalities [82].

A two-way communication system for Mexican Sign Language in a medical context is presented, which utilizes two sensors: Leap Motion (for precise finger motion capture) and Microsoft Kinect v2 (for body and upper body pose tracking). The classification uses the DTW algorithm for temporal sequence alignment and k-NN for gesture recognition. The system is designed for limited medical situations and shows high accuracy in sensory fusion situations [83].

#### 3.4.2. Sensor-Based Multimodal Fusion Systems

**Figure 5:**
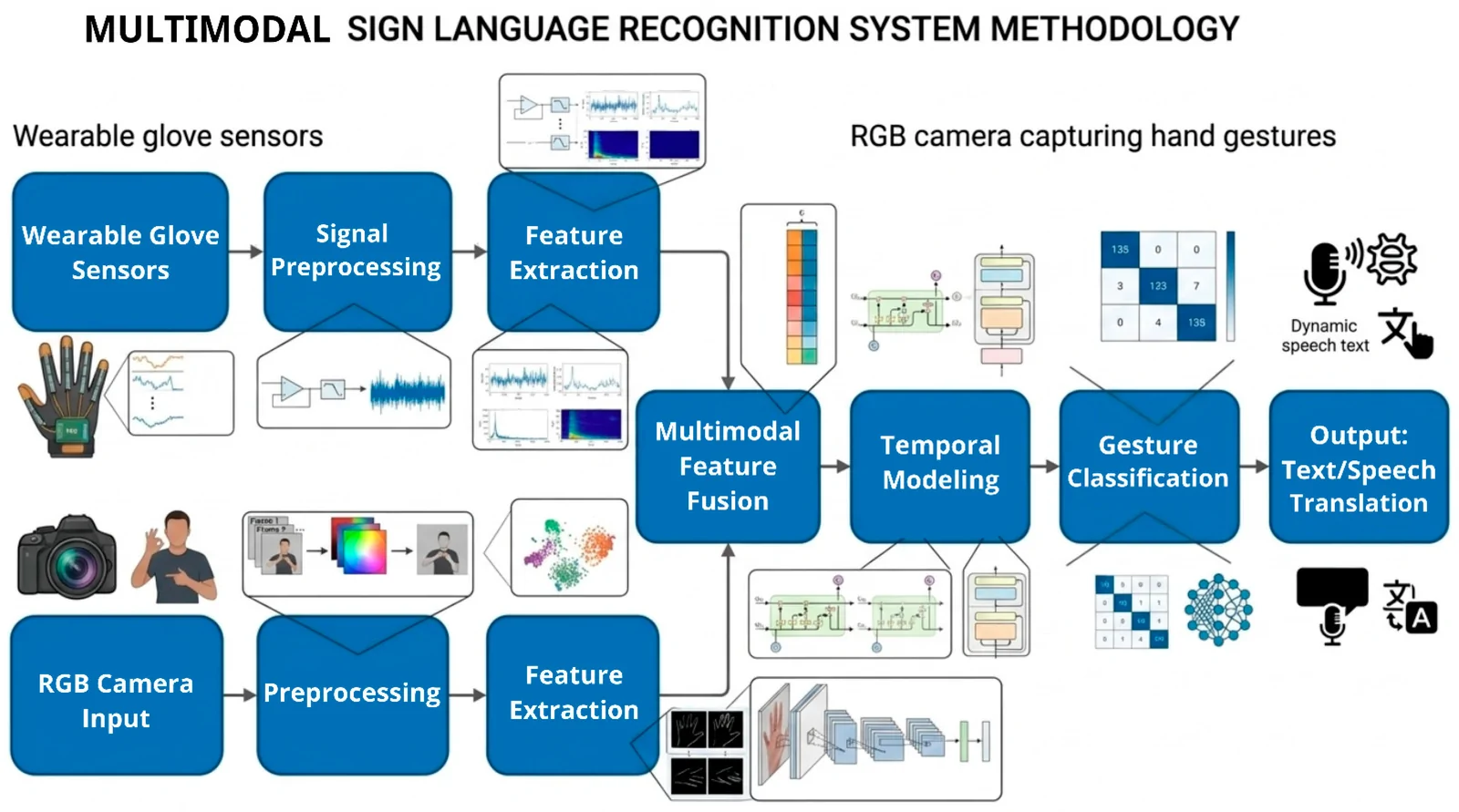
General architecture of a multimodal SLR.

Figure 5 shows an SLR system based on multimodal data fusion. Such an approach is widely considered in the current literature as an effective means of increasing recognition accuracy by combining complementary sources.

In general, such a system implements a 2-thread data processing pipeline and parallelizes signals of different nature.

1. *Data acquisition and pre-processing.* Input data is obtained from wearable sensor devices (e.g., sensor-equipped gloves) or RGB cameras. The sensor signals undergo standard filtering procedures, such as noise reduction and normalization, while the video stream is processed using image enhancement and color space conversion techniques to improve robustness to external conditions.
2. *Feature extraction and fusion.* For each modality, separate feature vectors are generated, reflecting both the kinematic characteristics of movements and the visual features of gestures. Next, multimodal feature fusion is performed, allowing for the formation of a common data representation and the preservation of both spatial and dynamic characteristics of gestures.
3. *Temporal modeling and classification.* The combined features are fed into temporal processing models (e.g., recurrent neural networks or transformers) designed to account for the sequential nature of gestures. The final stage is classification, which allows the recognized gestures to be converted into text or synthesized speech.

The use of such architectures allows for the compensation of limitations of individual modalities, ensuring more robust and accurate sign language recognition, especially in real-time conditions and under complex external factors. This concept has been validated in several applied studies.

Using the measured dynamic time warp (DTW) fusion strategy, researchers in [84] demonstrated a a wearable SLR system that can integrate both bending and inertial sensors.

Let $X_{k}\;=\;\{x_{1}^{\left(k\right)},\ldots x_{T_{X}}^{\left(k\right)}\}$ and $Y_{k}\;=\;\{y_{1}^{\left(k\right)},\ldots y_{T_{X}}^{\left(k\right)}\}$ represent the time series obtained by the k-th sensory modality, where k∈{1,…,K} is compatible with a tilt sensor and an IMU.

The DTW distance k is defined by modality as:

(18) $$D_{DTW}^{\left(k\right)}(X_{k},Y_{k})\;=\;\underset{\pi }{\min}\sum_{(i,j)\in \pi }||x_{i}^{\left(k\right)}\;-\;y_{j}^{\left(k\right)}|{|}_{2},$$

where π denotes the optimal temporal alignment path between two sequences.

The weighted multimodal fusion distance is computed as:

(19) $$D_{\mathrm{fusion}}(X,Y)\;=\;\sum_{k=1}^{K}w_{k}\cdot D_{DTW}^{\left(k\right)}(X_{k},\;Y_{k}),$$

subject to:

(20) $$\sum_{k=1}^{K}w_{k}\;=\;1,\;w_{k}\geq 0,$$

The proposed method applies Dynamic Time Warping (DTW) independently to each sensor modality to in order to compensate for temporal variations in gesture execution speed. DTW aligns two time-series sequences by minimizing the cumulative Euclidean distance along an optimal warping path.

Any distance characteristic of the modality contributes to the final similarity score with the weighting coefficient $w_{k}$. These weights regulate the relative importance of bending sensor data (capturing finger articulation) and inertial data (capturing orientation and motion dynamics). The final recognition decision is obtained by comparing the input gesture against stored reference templates and selecting the class with minimal fused distance. This approach enables robust handling of variable execution speeds and improves multimodal integration compared to single-sensor DTW systems.

Despite its effectiveness, several limitations remain:

- Static modality weighting. For static modal measurements, the weights $w_{k}$ are predefined and fixed worldwide. However, each movement has a different additional movement element (finger articulation, orientation). Static measurement methods cannot adapt to the relevance of movement-dependent methods.
- Absence of learned feature representations. The method operates directly on raw sensor measurements without nonlinear feature learning. Consequently, it cannot capture complex articulation dynamics or hierarchical motion abstractions.
- No cross-modal interaction modeling. The additive fusion formulation:
  (21) $$D_{\mathrm{fusion}}\;=\;\sum_{k=1}^{K}w_{k}D_{k},$$
  assumes independence between modalities. In practice, finger bending and inertial motion are biomechanically correlated, and such dependencies are not explicitly modeled.
- Scalability limitations. The template-based classification requires comparison with all reference sequences. For C gesture classes and sequence length T, the computational complexity scales approximately as O(CT^2^), limiting large-vocabulary scalability.
- Limited inter-user generalization. Sensor-based systems are sensitive to hand size, glove positioning, and motion style. Since DTW does not learn invariant representations, cross-user robustness remains insufficiently addressed.

These constraints demonstrate that adaptive multimodality weighting mechanisms, deep chain representation learning, and cross-modal attention-based architectures can significantly enhance scalability, reliability, and generalization performance in wearable SLR systems.

The system in the study combines the signals of flexible sensors and accelerometers into a single representation of the features presented in the classification [85].

Flexible sensors provide real-time measurements for finger flexion and accelerometer movement dynamics in order to create an overall feature vector containing the articulation and movement information. A support vector machine (SVM) will generate decision boundaries based on the combined signal using kernel functions based on the two-dimensional data. The SVM’s primary goal is to accurately classify short segment motions or static motions by maximizing the separation threshold parameter data between two class representations; this method lends itself to a moderate amount of processing complexity and provides a system optimized for efficient real-time SVM training and execution.

Although the wearable device shows signs of effectively finding gestures in real time, there are several constraints from a theory and practice standpoint. To begin with, the fusion of features relies on concatenating vectors, but that does not explicitly show how the flex sensor or accelerometer interact and what the correlation between them is. Secondly, the SVM classifier uses fixed-length feature vectors and therefore does not inherently provide a means for identifying any temporal awareness when a gesture is done dynamically, limiting its ability to find sequential articulation. Also, with the kernel-based system, the computational cost of finding a kernel-based classifier and additional support vectors used for finding various kernels does not scale well with large training datasets and with larger gesture vocabularies. Finally, this system does not learn hierarchical feature representations; therefore, it has limited generalization ability across users having different performance styles, hand shapes, or methods of placing their sensors. These characteristics indicate that sequence-aware deep learning architectures and adaptive multimodal fusion methods can provide more robust and scalable wearable SLR systems.

Gloves with inertial and bending sensors are used to train and assist people with hearing impairments by providing real-time gesture recognition [86]. In a subsequent study [87], gloves with 5 flexible sensors and a 2-axis accelerometer demonstrate high stability in real time.

Researchers [88] have combined several IoT sensors mounted on a body to recognize movement patterns with integration at the functional level.

Temporal aggregation over a gesture sequence of length T produces a global feature vector:

(22) $$x\;=\;\frac{1}{T}\sum_{t=1}^{T}x_{t},$$

Various IoT sensors that collect motion using more than one method create a multidimensional data input, which can then be processed independently as feature vectors. The feature vectors from each motion channel will then be combined into one feature vector using concatenation before the final gesture detection stage. Temporal data will also be condensed and non-temporally aggregated into one big descriptor that describes the gesture being detected. Finally, the fused feature vector will be classified into a gesture class using a discriminative classifier that creates posterior probability estimates for each gesture class. The intended outcome of this architecture is to have greater robustness by taking advantage of complementary motion information through body location (joint) using multiple IoT sensors. Additionally, the use of a fusion-based recognition strategy has been shown to improve gesture recognition compared to recognition systems that only use one sensor per motion (hands using a finger, tripod).

While there are benefits to multimodal feature-level fusion, such as increasing the accuracy of gesture recognition, this method has limitations. A major drawback is that the concatenation fusion method assumes that the sensor devices used to capture the motion data are completely independent and do not model any biomechanical dependencies that may exist between the body parts. Since temporal aggregation is performed through averaging the frames, there is a potential risk of losing fine-grained detail of the sequence, including transitions. Additionally, the framework for classification was performed using either handcrafted or manually extracted variables rather than learned hierarchical representations, which limits the ability for gestures to be recognized in different individuals or movement types. The ability to scale to more complex gesture vocabularies and be able to operate robustly in real-world environments is an area where additional work still needs to be done. Therefore, future work on developing adaptive, multimodal fusion methods and deep learning models that consider the sequence of gestures may improve the robustness and representation of the IoT gesture recognition systems used.

In the 2024 study by Sümbül [89], a wearable glove for gesture recognition and reproduction was developed using five flexible finger sensors and a three-axis accelerometer. The dataset includes 15 ASL gestures and the digits 1–5, with 5204 data points converted to Pulse Width Modulation (PWM) values for feature unification. A Multi-Layer Perceptron Feed-Forward Neural Network (MLPFFNN) with backpropagation training was used for recognition. The system ensures gesture reproducibility, is efficient with small datasets, and is low-cost to manufacture.

Multimodal touch gloves with stretchable sensors and microstream sensors for 3D hand trajectory demonstrate versatility and economy of training data [90]. Gloves with conductive knitwear and an IMU analyze dynamic gestures with LSTM, providing autonomy and energy efficiency [91]. This article presents a wearable touch glove and an Android app for real-time sign language recognition and pronunciation [92]. A combination of tensor-resistive sensors and an IMU with a hybrid CNN–LSTM architecture demonstrates high performance in recognizing ASL letters and words, achieving accuracy over 99% [93].

In the following work [94], the authors combine IMU signals, sEMG, and facial expression tracking to achieve multimodal sign language interpretation.

The system has a collection of complementary modalities: motion and orientation of the hand are recorded with the motion sensor; the activation of muscles is recorded with the sEMG sensors, and the non-manual cues, which are our facial movements, are recorded with the facial tracking system. All the data generated by the three modalities can be combined into a single data vector (feature vector). The feature vectors are processed through a Recurrent Neural Network (RNN) to take into account the temporal relationship between gesture sequences. By including both manual and non-manual components, the model is attempting to represent the entire linguistic structure of ASL. The final layer of the RNN uses a softmax function to output posterior probabilities for translated output.

Despite making significant advances in sign language translation through the use of a multi-modal approach, there are still limitations to the proposed framework. One limitation is that the current method of data fusion is based on simple concatenation and does not take into consideration how modalities interact with one another from a hierarchical or attention-based perspective. Additionally, while there are many reasons that the contributions of facial expressions and muscle activity may change throughout a given sign, there currently are no adaptive weighting mechanisms in place within the architecture that would allow for accounting for this variability. Another limitation of the functionality of recurrent networks is that they model only short-term dependencies of signs and may not adequately capture long-term dependencies and the complexity of the grammatical structure of ASL. Finally, the current implementation of the system assumes that all three modalities are in sync and consistently available from the sensors. However, in actual deployment, sensor latency, occlusion, or dropouts may cause the modalities not to be in sync or present; therefore, the models developed may be inaccurate. Additionally, cross-user variability in muscle activation patterns and facial expressions poses generalization challenges that are not fully addressed. These factors indicate that future research should explore adaptive cross-modal attention mechanisms, transformer-based sequence modeling, and robust multimodal synchronization strategies to enhance wearable sign language translation systems.

In the 2024 study by Salmankhah et al. [95], 5 flexible sensors and Persian End-to-End Sign Language Recognition (PenSLR) IMU are proposed to recognize sentences in Persian Sign Language. In the 2024 study by Begum et al. [96], a sensor glove with flex sensors, an accelerometer, and a gyroscope is proposed to recognize Bengali Sign Language. The Dataglove glove with 16 nine-axis IMUs and CNN-BiLSTM provides high accuracy [97].

Multimodal synthesis and sequential modeling were implemented in [98] to implement continuous dynamic gesture recognition.

The system performs feature-level fusion by concatenating multimodal inputs at each time step. This unified representation captures complementary information from different sensing channels. A Long Short-Term Memory (LSTM) network captures temporal relationships throughout a sequence and addresses the identification of moving gestures as they change over time. Continuous identification (vs. isolated classification) requires modeling the transitions between one sign and another and retaining the articulation dependent on context. The probability of each class is produced by the softmax layer at each time point, while the decoding of the input’s movement stream identifies the most likely series of gestures that corresponds to the input.

Although the proposed framework improves upon continuous gesture identification through multimodal fusion and recurrent modeling, it cannot fully support contextual understanding or scalability due to the structural limitations resulting from the lack of illustrating natural sign language’s hierarchical and long-range relationships within its integration and temporal decoding mechanisms.

In the 2023 study by Li et al. [99], a wearable multi-sensor system is used that combines sEMG muscle activity signals and accelerometer data, which are fused using a hidden Markov chain model to recognize gestures with an accuracy of 90.41%. In the 2024 study by Sahm et al. [100], a wearable glove for ASL gesture recognition was proposed using five slide potentiometers and two force sensors (FSR) to record finger movements and pressing force, followed by gesture classification using a neural network in real time.

The system proposed in [101] combines multimodal wearable sensors and deep neural networks for SLR.

The framework integrates multiple wearable sensor modalities and applies deep learning for feature extraction and temporal modeling. By concatenating multimodal signals and applying sequential neural architectures, the model learns hierarchical motion representations directly from sensor streams. By using end-to-end representation learning rather than using standard methods for classifying, machine learning classifying produces a positive increase in recognition performance when using classifiers.

These deep multimodal approaches provide greater representation learning than traditional fusion modes of integrating features using only concatenation without modeling the relationship of one kind of feature to another. Furthermore, there is no use of an adaptable attention weight to re-evaluate the importance of modalities depending on context when recognizing gestures. In addition, using recurrent-based approaches to modeling will have difficulty capturing long-term history or the proper grammar of extended gestures. There are also research opportunities with regard to using larger vocabulary sets, dealing with sensor noise and considering normal variations across individuals. Therefore, multimodal systems represent significant innovations, combining multiple modalities, adapting to motion variability, and using attention and graph structures to extract important features. The main limitations include high computational complexity, dependence on the quality of sensors and cameras, hand occlusion, data noise, and limited vocabulary. These limitations are addressed by modality fusion, attention mechanisms, reinforcement, and transfer learning.

This reflects the constant evolution of sign language recognition systems, from simple communication gloves to multimodal neural network systems. While single-modal systems (RGB only or sensor only) have reached their limits in occlusion and variable lighting conditions, the future of SLR systems lies in hybrid solutions capable of intelligent data fusion (Feature Fusion) and adapting to the user’s natural, continuous speech.

## 4. Evaluation Metrics

The performance of sensor-based sign language recognition systems is evaluated using standard classification metrics and sequence analysis. The choice of metrics depends on the type of task (recognition of static gestures, dynamic sequences, or continuous signed speech) and the models used. The most common metric is accuracy, which indicates the proportion of correctly classified gestures out of the total number of observations. However, in tasks with unbalanced classes, additional metrics such as precision, recall, and F1 scores are used to more accurately assess the recognition quality of individual gesture classes.

The Word Error Rate (WER) is widely used in continuous sign language recognition and sequence processing tasks, and the Sign Language Error Rate (SER), which is also used for Ga language, is used. These metrics take into account errors during insertion, deletion, and substitution, and carefully assess the quality of recognition at the sequence level.

In real-time systems, timing characteristics such as latency and throughput play a crucial role in determining the feasibility of a model. Computational costs such as memory usage and power consumption are also considered, especially for wearable sensor devices. Sensor systems using IMUs, EMGs, and other devices are also evaluated for their resilience to noise and signal fluctuations. This is an important factor when operating in real-world conditions.

Combining metrics such as accuracy, sequential recognition quality, and computational efficiency allows for a comprehensive assessment of the performance of modern sign language recognition systems.

## 5. Comparative Analysis of Methods

Based on methodological approaches, three main groups are distinguished: conventional methods, machine learning methods, and deep learning methods. This classification reflects the evolution from approaches based on manual feature creation to fully automated feature learning using deep neural networks. Hand gesture recognition analyzes hand poses or trajectories to interpret user-spoken information and convert it into understandable text or actions for the system or human.

Within conventional methods for recognizing static gestures, approaches based on template matching and general-purpose classifiers are widely used. Such systems are characterized by simplicity of implementation, low computational complexity, and real-time operation. However, they are limited by a small gesture vocabulary and weak generalization ability, as shown in Table A1 (see Appendix B), which summarizes the literature review on sign language recognition based on conventional methods.

For dynamic gestures, temporal sequence alignment methods are used, the most common of which is dynamic time warping (DTW). This approach accounts for variability in gesture execution speed and ensures robust comparison of temporal sequences of movements. For example, in [84], DTW is used in conjunction with weight alignment of Flex and IMU sensor signals for real-time gesture recognition. In the 2023 study by Galván-Ruiz et al. [46], DTW is used in conjunction with a 3D skeletal hand model (Leap Motion) to recognize a large vocabulary of gestures, achieving high accuracy and robustness to speed variations. Furthermore, in [83], a hybrid DTW-kNN system is proposed, targeting medical applications, where DTW is used for temporal alignment and kNN for gesture classification.

Gesture segmentation plays a crucial role in recognition systems, as it directly impacts the accuracy of feature extraction and subsequent classification. Effective gesture boundary detection improves overall system performance.

Machine learning (ML) algorithms are widely used in hand gesture recognition due to their ability to make decisions based on training data and identify patterns in sensory signals [38,85]. This approach provides a balance between computational efficiency and recognition accuracy in real-time systems.

The most common classification methods include support vector machines (SVM), k-nearest neighbors (KNN), linear discriminant analysis (LDA), random forests, and ensemble models. For example, refs. [37,38,86] showed that combinations of SVM and KNN, as well as Random Forest, provide high gesture recognition accuracy when using sensory data from Flex-based gloves and IMUs. Ensemble machine learning methods such as Random Forest, SVM, and Naive Bayes are used for processing EMG and IMU signals, providing high system speed and scalability, as presented in [85]. However, such systems remain dependent on the quality of devices and are sensitive to sensory data noise.

Neural network models, including MLP, are also used for static gesture recognition, especially in systems with limited computational resources [87]. Such solutions achieve low implementation costs, but are limited to working with static gestures. Furthermore, hybrid approaches combining multiple machine learning algorithms (SVM, KNN, MLP) demonstrate higher robustness to gesture variability and are used in tasks with more complex recognition scenarios [88]. However, such systems may have difficulty distinguishing between similar gestures. Several studies have examined systems using magnetic sensors and positioning methods combining SVM and k-NN to improve robustness to shielding and spatial variations [27]. This approach provides robustness but is susceptible to magnetic noise. A comparative analysis of different machine learning algorithms, summarized in Table A2 (see Appendix B), shows that their performance varies depending on the sensor type, gesture complexity, and computational requirements. Furthermore, deep learning is a set of learning methods used to extract and represent data with complex structures and model nonlinear dependencies [29,31].

Table A3 (see Appendix B) shows key studies covering everything from simple gesture recognition (letters, numbers, and words) to more complex tasks such as recognizing sequences and sentences in various sign languages.

In simple deep learning models, MLPs and artificial neural networks (ANN) are widely used to classify static gestures [29,31,89,100]. These methods are characterized by low computational complexity and can be implemented in real time, but they are limited by the inability to model the temporal dynamics of gestures.

Recurrent neural networks (RNNs), including LSTMs and GRUs, are actively used for processing temporal sequences. Studies [90,91,95] demonstrated that LSTM and GRU models effectively model the temporal dependence of gestures and support continuous sign language recognition. Accordingly, BiLSTM and BLSTM architectures were used to improve robustness to sequence variations [30,98].

CNNs are widely used to extract spatial features from images and video data [33,93,96]. By combining CNNs and LSTMs to build a hybrid model and simultaneously extracting spatial and temporal features, the accuracy of dynamic gesture recognition was significantly improved. To achieve high-accuracy gesture alignment, more complex architectures are used, in particular, CNN-BiLSTM-CTC and Transformer models. For example, studies [35,94] note that these models support sentence-level recognition and sign language-to-text translation in near real time. Furthermore, attention-based mechanisms are being actively implemented to achieve high recognition accuracy by identifying the most relevant features [97,99]. These approaches enable the efficient processing of multisensory and multimodal data, such as IMU, sEMG, and RGB video.

Recent studies have employed methods for modeling skeletal data and complex spatiotemporal dependencies using graph neural networks (GCNs) and transformers [59,63,66]. These methods demonstrate high accuracy but require significant computational resources.

More advanced systems combine multiple architectures such as CNNs, GCNs, transformers, and GANs to perform end-to-end sign language recognition, translation, and generation tasks [67,71]. These models achieve high accuracy but are characterized by high computational complexity and rely on large amounts of data. The comparative analysis of the presented methods, given in Table A3 (see Appendix B), demonstrates the transition from simple neural networks to complex multimodal architectures that provide high-precision processing of both static and dynamic gestures.

Table 2 aggregates the accuracy figures reported throughout Section 3 to allow direct cross-method comparison. Deep learning approaches evaluated under controlled, single-signer or curated conditions consistently report accuracies at or near ceiling (98–100%, e.g., [59,68,93]), whereas the same class of methods evaluated under more realistic or cross-domain conditions shows a marked drop: 82.66% for SLATN on the Pakistani Sign Language dataset [77] and 77% for event-based spiking-network recognition [45]. This spread illustrates why raw accuracy values cannot be compared directly across studies without accounting for vocabulary size, signer independence, and evaluation protocol, as discussed further below.

Three recurring problems underlie the accuracy figures reported above. First, cross-user generalization fails primarily because wearable and vision-based systems are highly sensitive to sensor placement, hand size, and individual signing style—variability that most reviewed systems do not explicitly model, which is why accuracy consistently drops when the same architecture is tested on new users (e.g., 99.17% → 96.67% in [42]). Second, static and continuous signing require fundamentally different temporal treatment: static-sign classification is a single-frame recognition problem, whereas continuous signing requires segmentation of co-articulated, boundary-less gesture sequences, which is why methods reporting near-ceiling accuracy on isolated signs (e.g., 98.8% in [44]) show substantially lower accuracy on continuous sequences (86.3% in the same study). Third, accuracy figures are not directly comparable across studies because they depend on vocabulary size, number of signers, and evaluation protocol—a 15-class, single-signer benchmark and a 2000-class, multi-signer benchmark cannot be meaningfully ranked on accuracy alone, which is why this review reports accuracy alongside these contextual factors wherever possible (Table 2) rather than treating it as a universal ranking metric.

## 6. Overview and Contribution of Collected Datasets

### 6.1. Research Directions in Existing Sign Language Datasets

One of the most significant challenges encountered in the development of sign language interpretation systems is visualizing gesture content for low-resource sign languages. One way to create a simple visualization system is to use a computerized video dictionary, compiled by a human. In such a system, words are presented as individual video fragments, and expressions are formed from these fragments. Despite the obvious advantages of simplicity, such systems have significant drawbacks. The main one is that gesture synthesis is not continuous, but rather consists of a sequence of isolated actions. Another method is to use a computer model of a person (Avatar). In this case, repetitive movements flow smoothly into one another, which is very effective for the perception of people with hearing and speech impairments.

A sign dictionary is a reference book containing the characteristics of gestures and their meanings. A sign language dictionary helps sign language learners learn new gestures and learn to use them correctly in conversation. A sign dictionary can also be used to translate simple words and phrases into sign language, so that people with hearing impairments can better understand what their interlocutors are saying and participate in communication.

Overall, a sign dictionary is an important tool for reducing barriers between people with hearing and speech impairments. Complex sign vocabulary can range from simple gestures like “hello” or “goodbye” to complex gestures such as medical, scientific, or technical terms. Sign dictionaries can also be used to create accessible communication environments for people with hearing and speech impairments in the community. For example, sign dictionaries or signs can be installed in public spaces such as airports, train stations, and hospitals to help people with hearing and speech impairments better navigate new places and communicate with staff.

**Figure A1:**
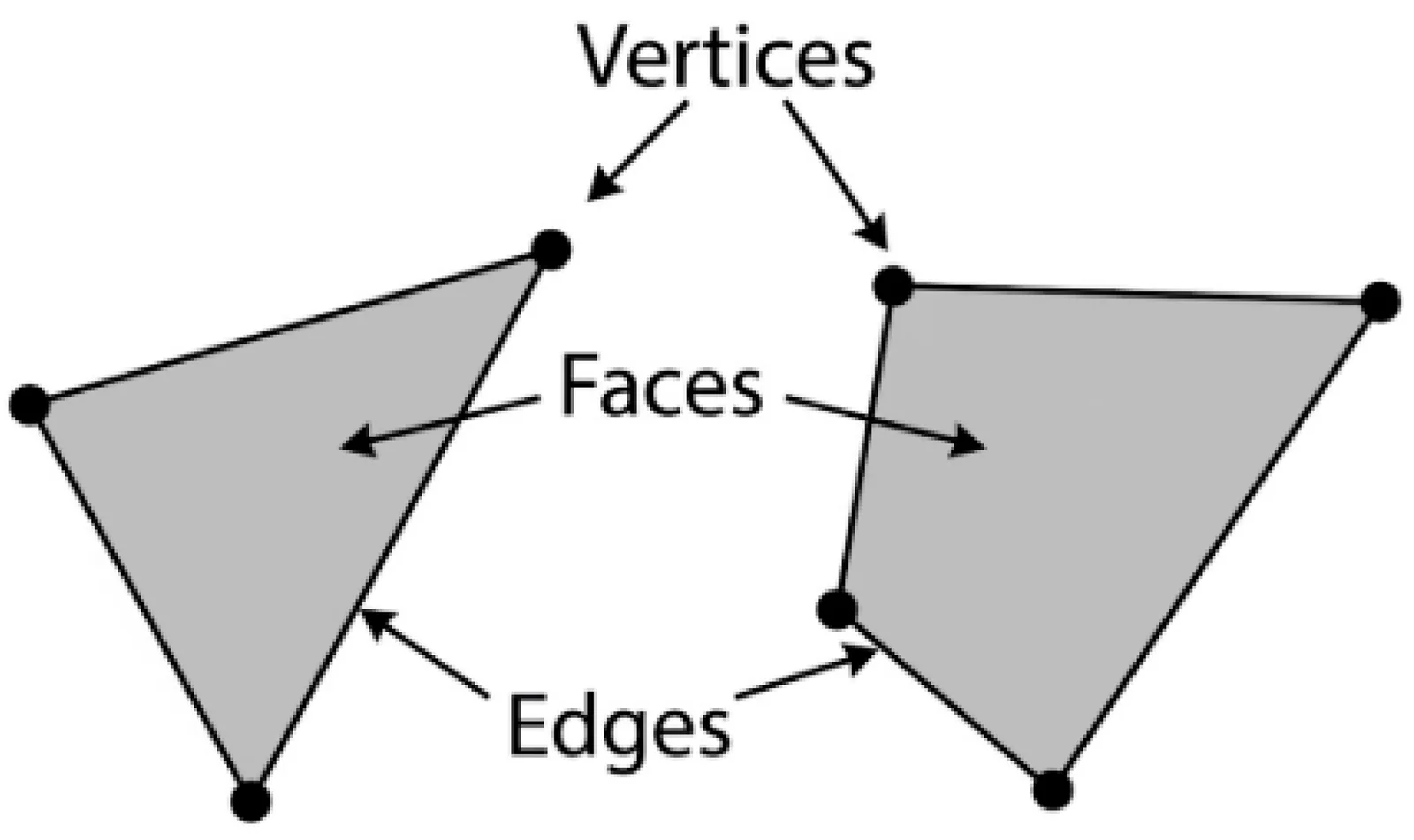
Schemes follow the same formatting.

**Figure A2:**
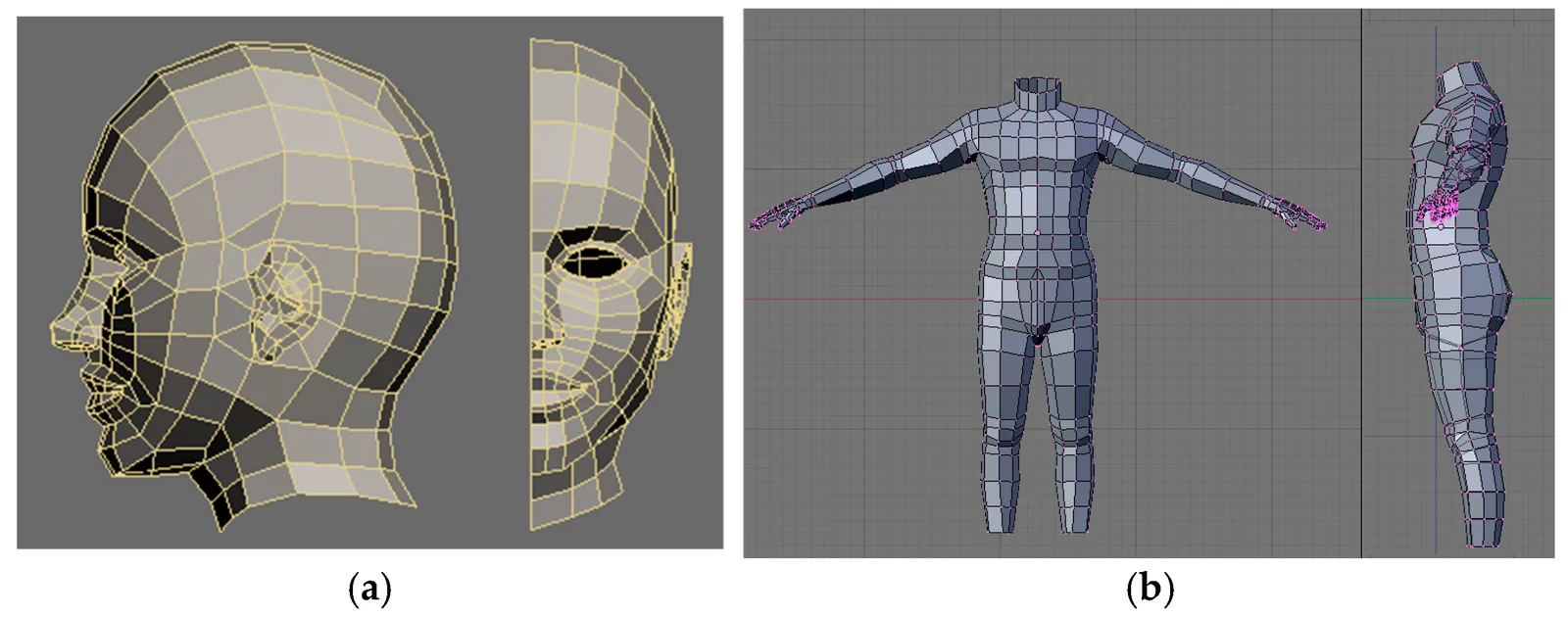
Creating a polygon model: (**a**) head model; (**b**) body model.

**Figure A3:**
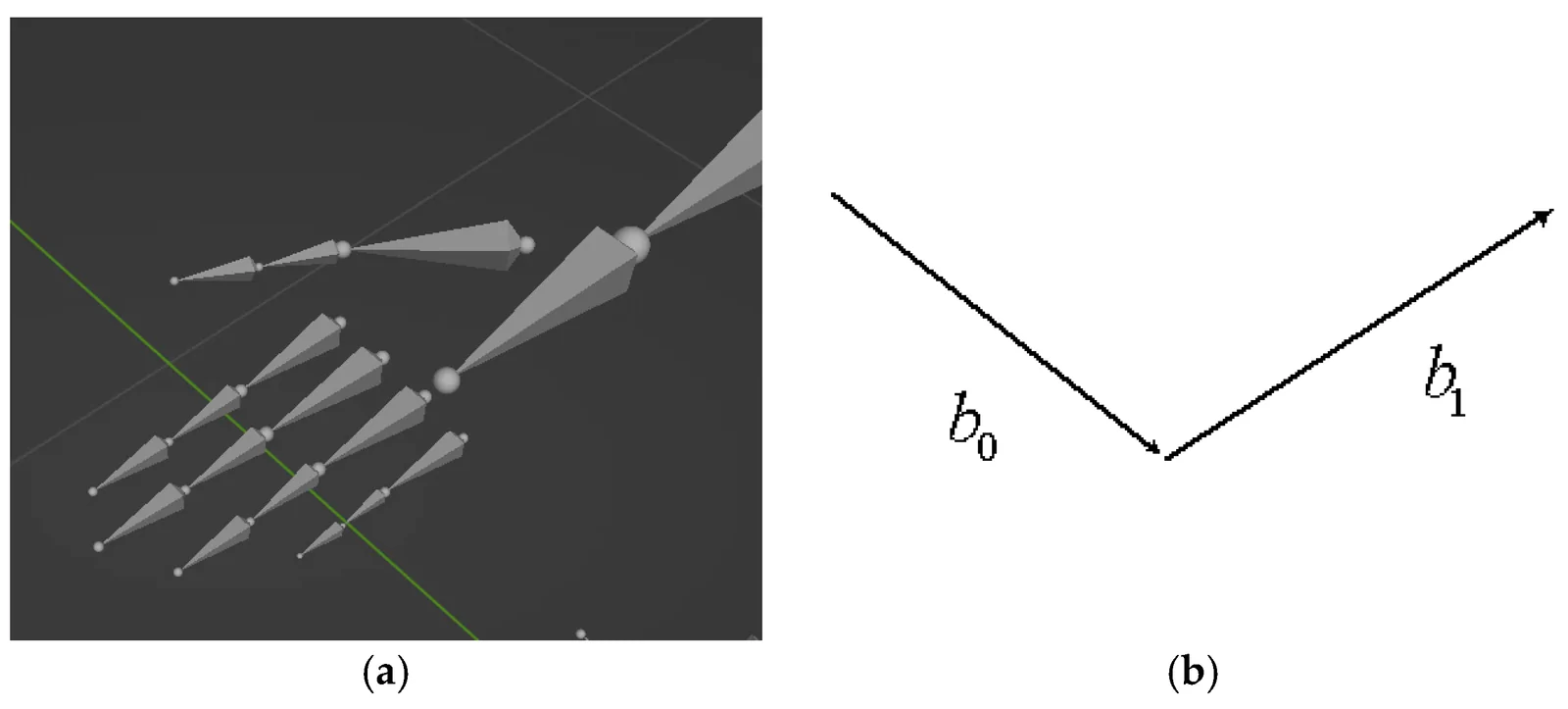
Example of bones: (**a**) hand bone representation extracted from the hand model; (**b**) bone vector defined between two connected joints ($b_{0}$ and $b_{1}$).

**Figure A4:**
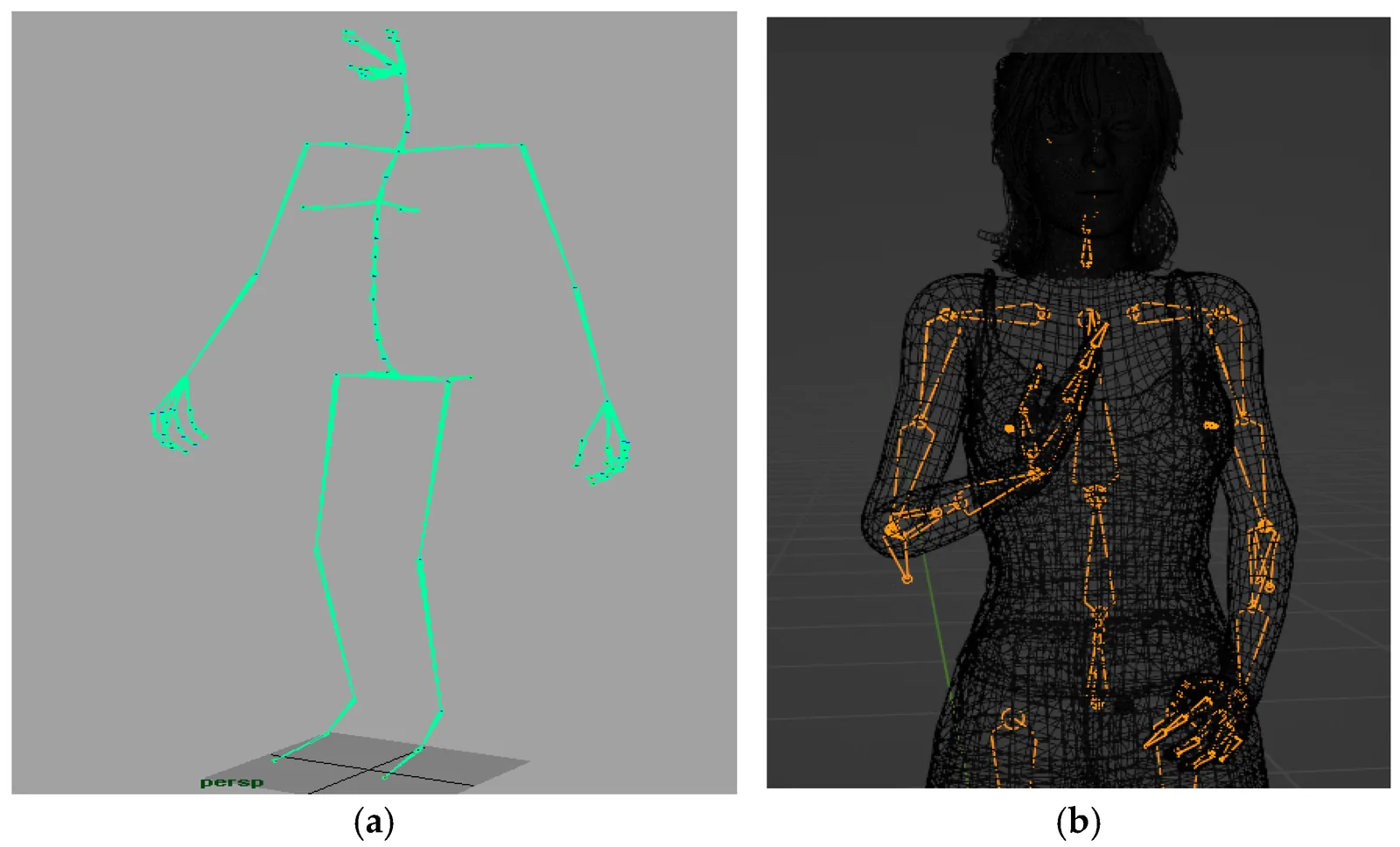
The built skeleton: (**a**) generated skeleton; (**b**) skeleton applied to the 3D human model.

**Figure A5:**
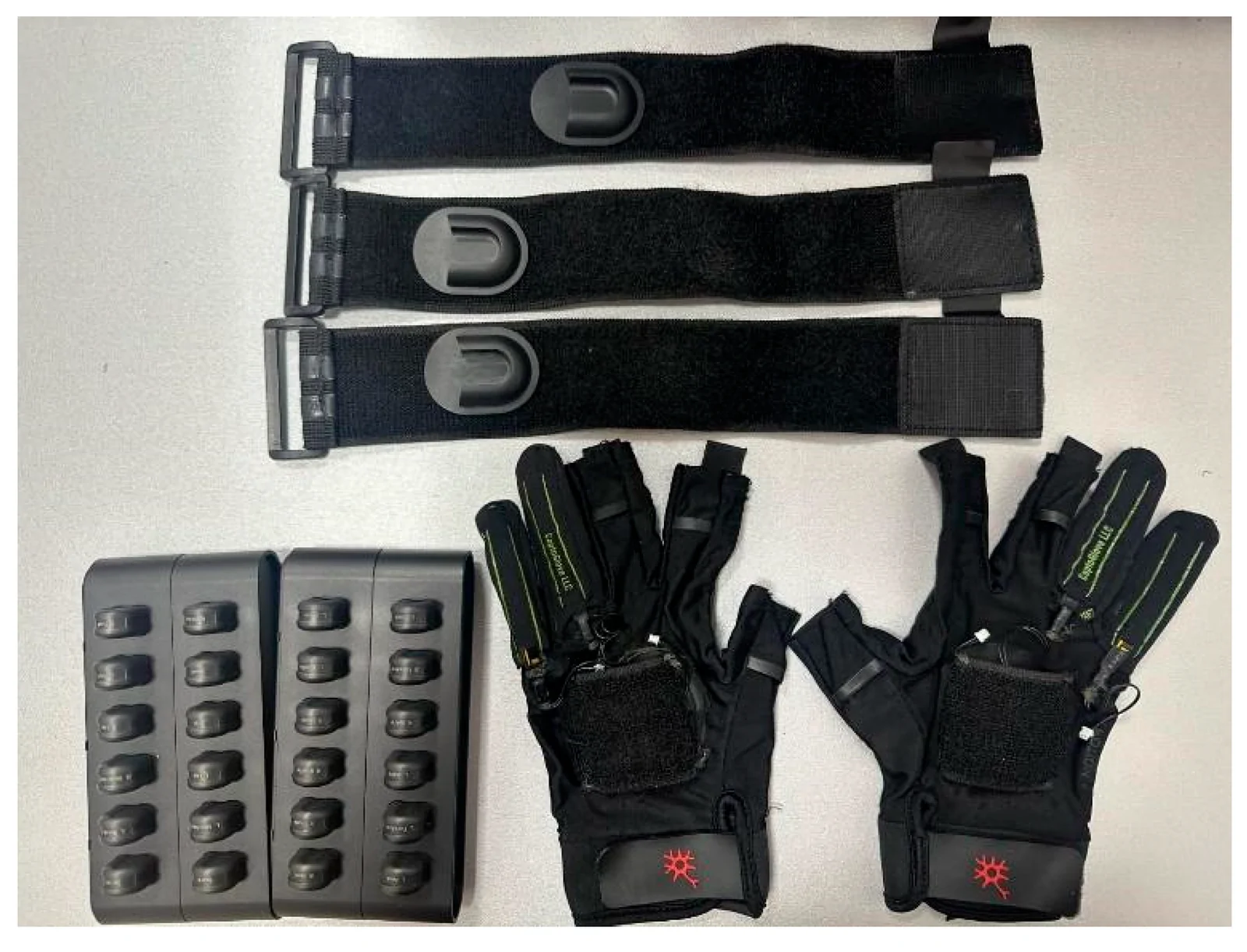
Sensors for capturing arm and upper body motion.

**Figure A6:**
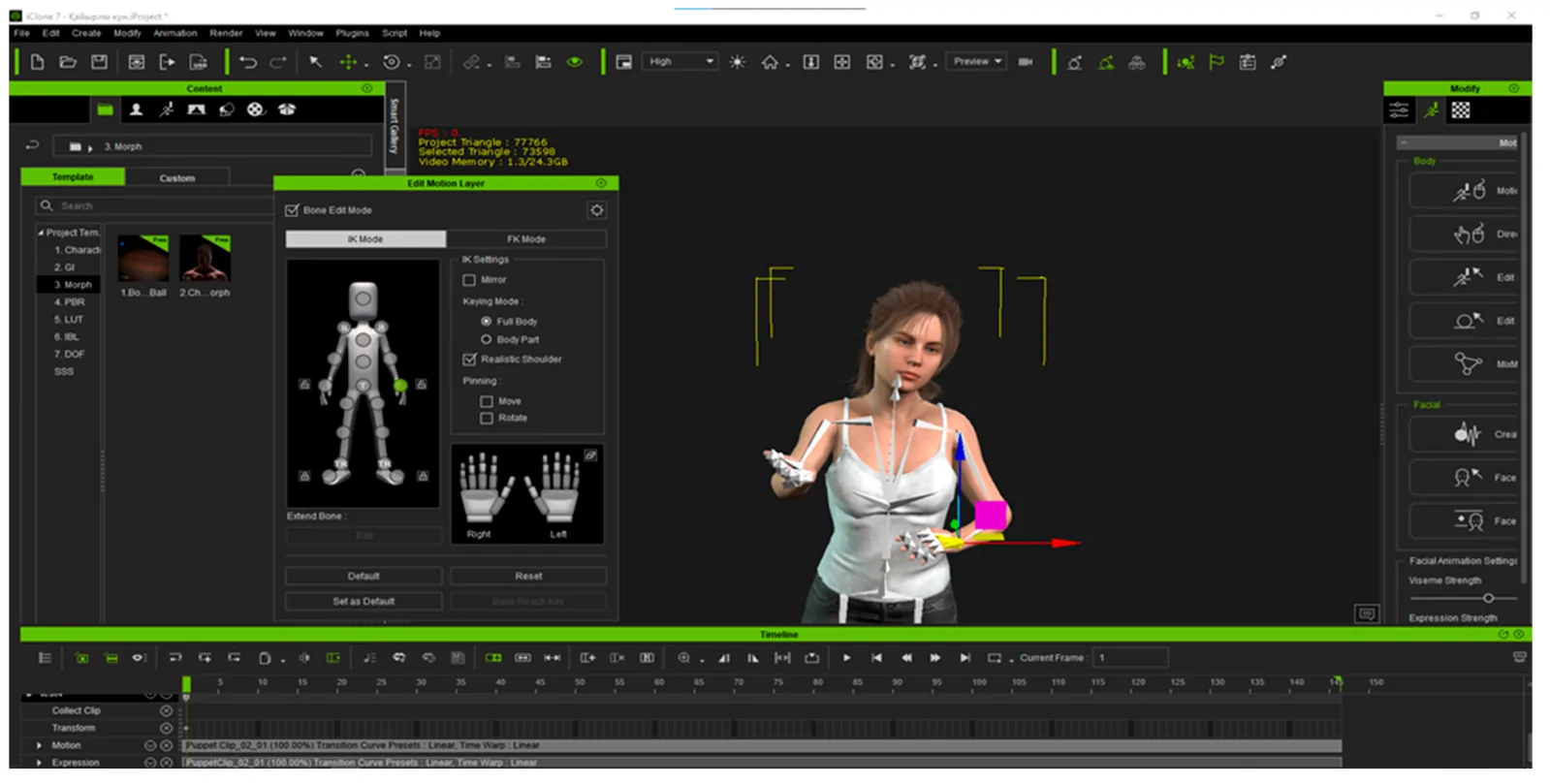
iClone interface for 3D animation and real-time rendering.

**Figure A7:**
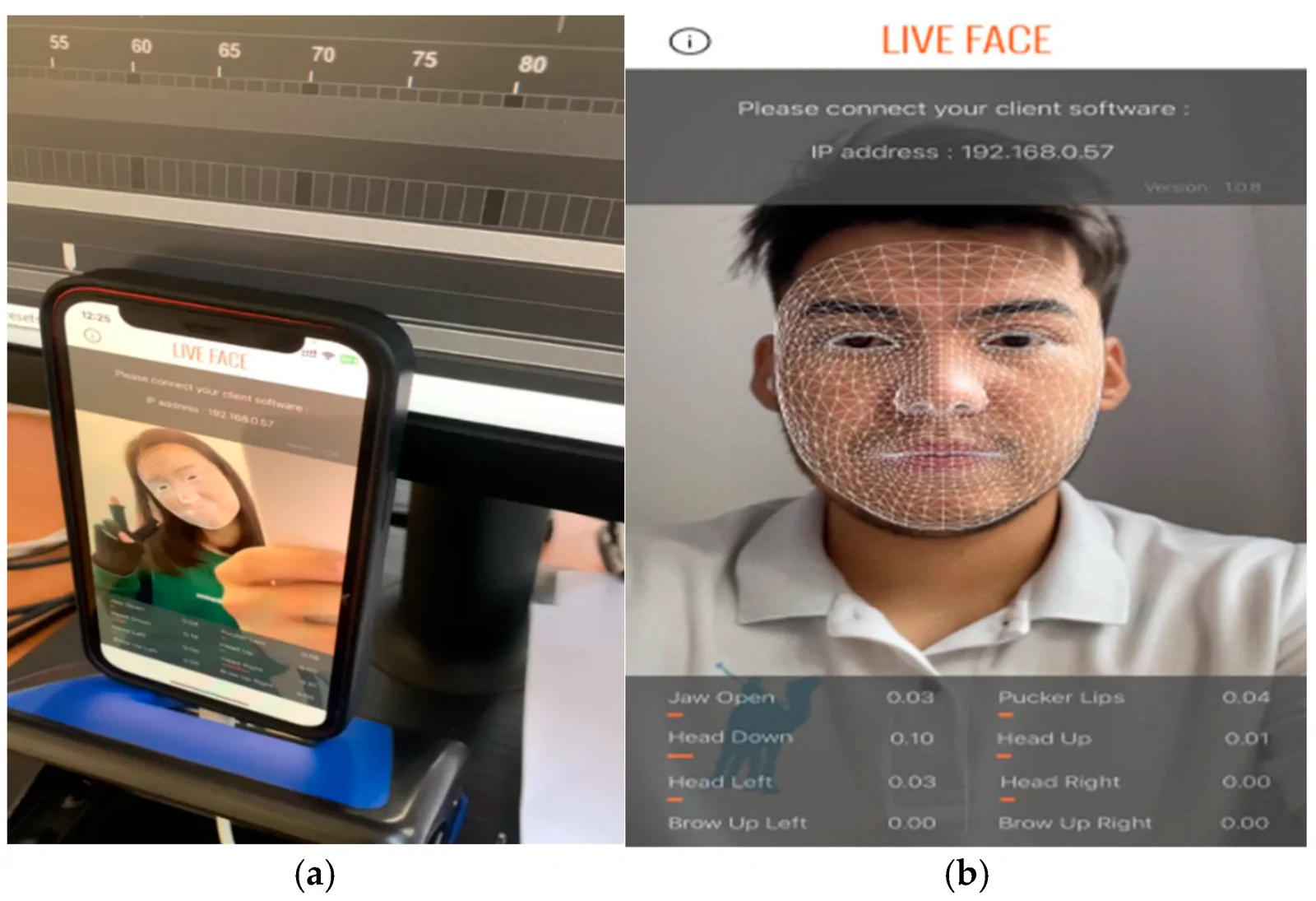
iClone Live Face: (**a**) mobile application; (**b**) facial tracking.

**Figure A8:**
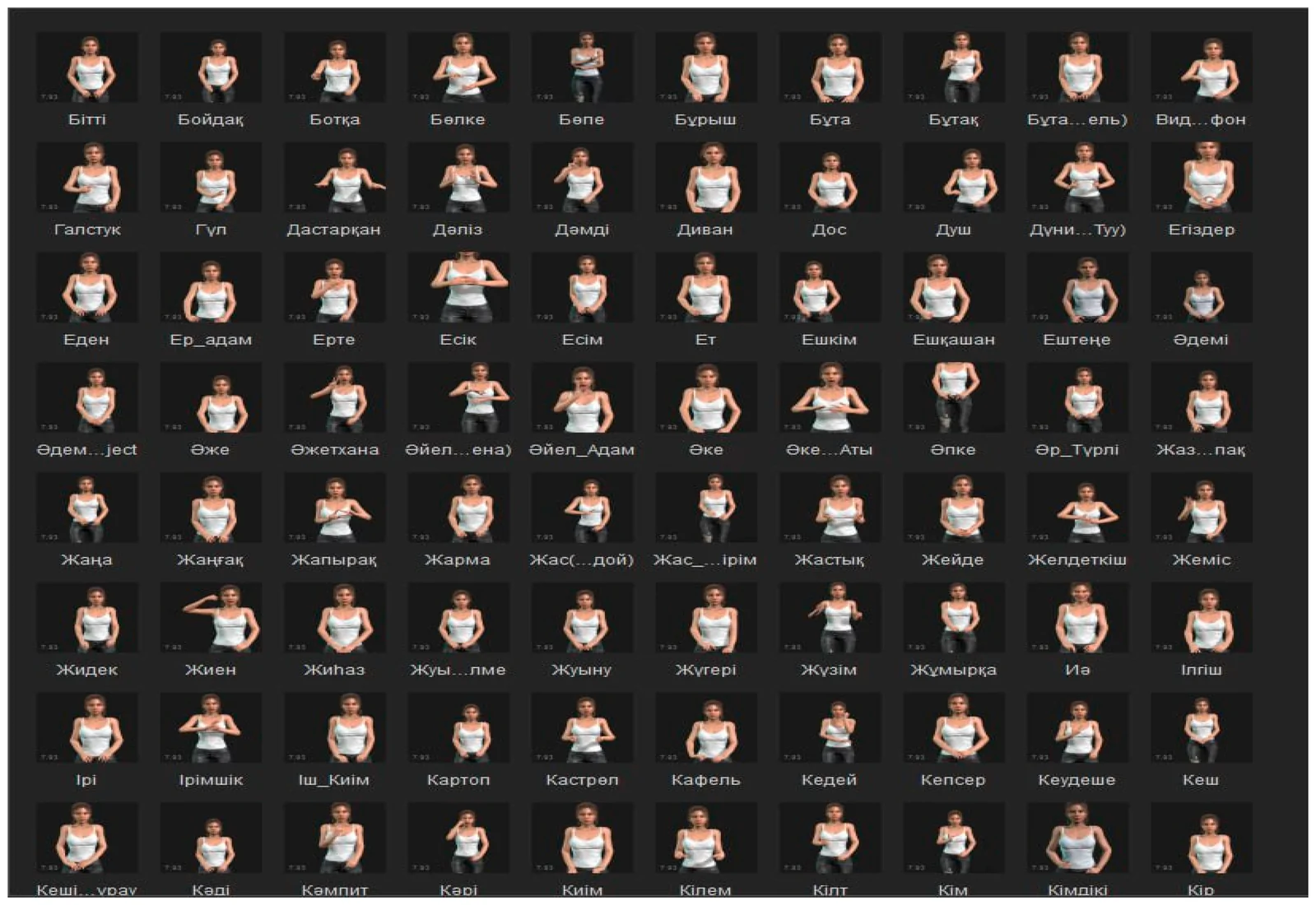
Animation Dictionary.

**Figure A9:**
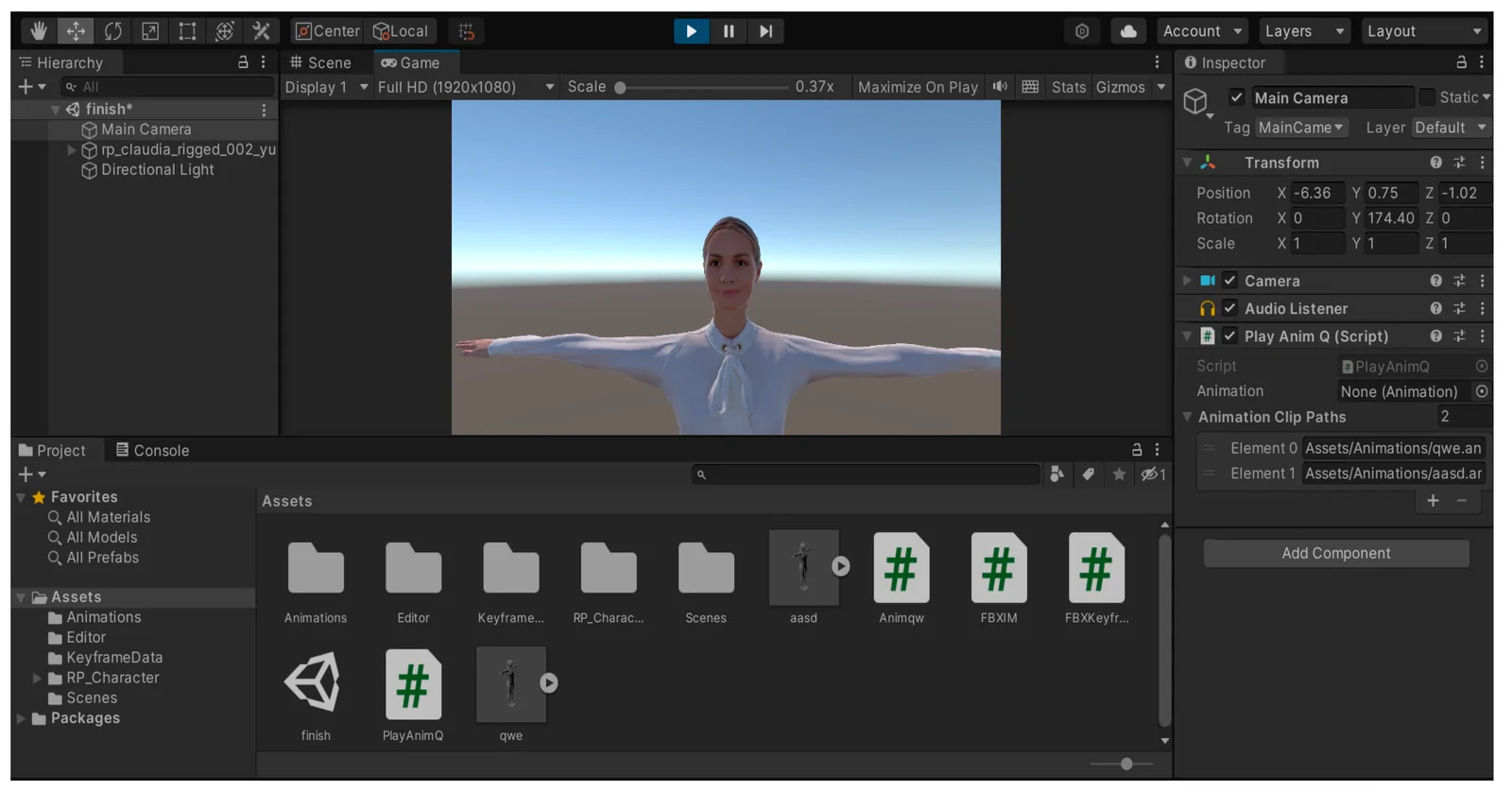
Unity interface.

**Figure A10:**
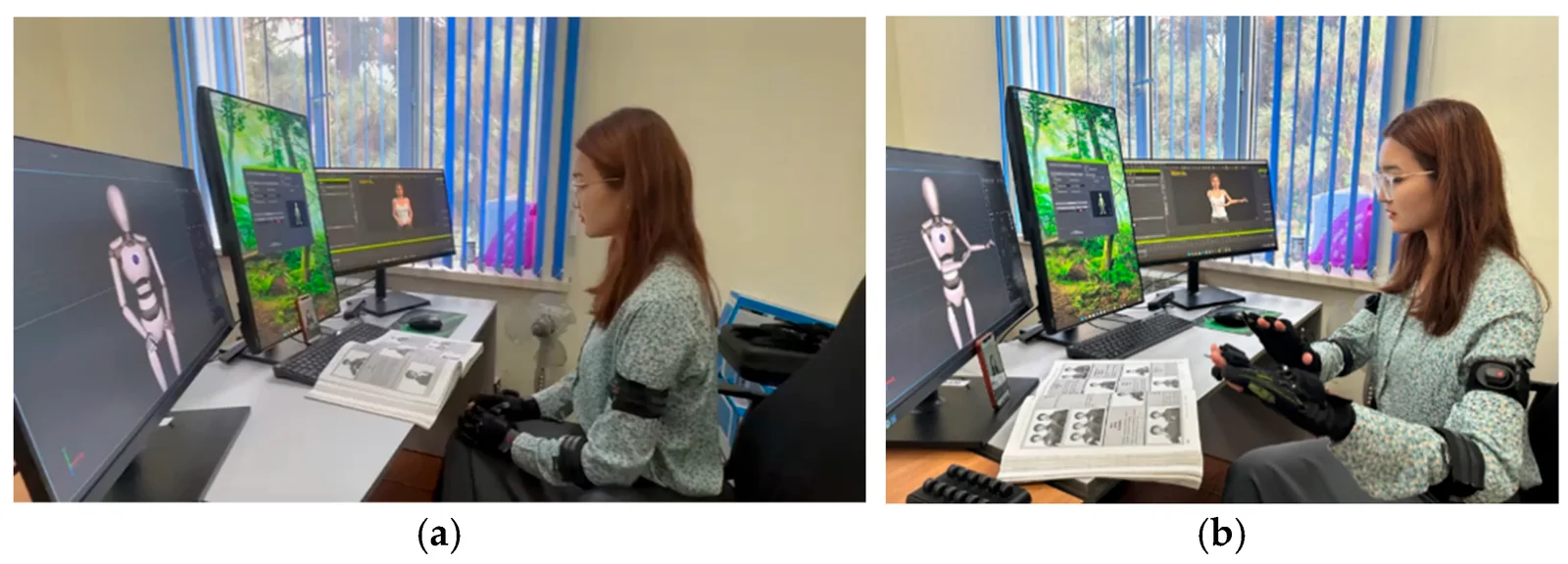
The process of creating a dictionary: (**a**) avatar preparation; (**b**) sign recording.

Creating an avatar-based visualization pipeline for sign language involves four general stages (illustrated in Figure A1, Figure A2, Figure A3, Figure A4, Figure A5, Figure A6, Figure A7 and Figure A8 of Appendix A): (1) anatomical modeling of a 3D human figure with realistic joint proportions, represented as a polygon mesh of vertices, edges, and faces (Figure A1 and Figure A2); (2) rigging—building a hierarchical bone structure (Figure A3), with the greatest concentration of bones allocated to the hand and fingers to support natural gesture articulation (Figure A4), since inadequate hand rigging is the most common cause of unnatural-looking gesture animation; (3) motion capture, using the Perception Neuron (Noitom Ltd., Beijing, China) wearable inertial motion capture system with optical or inertial sensors to record hand and body movement (Figure A5) alongside dedicated facial-capture systems using iClone 7, version 7 (Figure A6 and Figure A7), since facial motion (non-manual markers) must be captured simultaneously with manual gestures to preserve grammatical meaning; and (4) mapping recognized or annotated signs to corresponding avatar animations compiled into a sign dictionary (Figure A8), rendered and integrated into an interactive environment using a real-time engine such as Unity 2022 LTS version (Figure A9 and Figure A10). This pipeline is directly relevant to the low-resource dataset discussion in Section 6.2: it is the same avatar-generation process used to produce the paired RGB-plus-avatar sample structure described for AKSLD-2000, and represents a practical way for low-resource sign languages to extend a modest recognition-oriented video corpus into a dual-purpose resource supporting both recognition and avatar-based translation output, without requiring a proportionally larger raw video collection effort.

Given the central importance of data quality in sign language data processing, developing robust sign language recognition (SLR) systems is significantly hampered by the lack of representative, large-scale, annotated datasets.

### 6.2. Collected Dataset Resources for KSL

The development of robust mirroring systems is significantly hampered by the availability of representative, large, annotated datasets. This limitation is particularly noticeable in resource-constrained sign languages, including KSL, and publicly available corpora remain lacking for these languages. Table 3 presents a comparative analysis of the selected sign language datasets considered in this review, covering key aspects such as sign language, number of sign classes, number of video samples, recording method, annotation granularity, number of sign language speakers, and accessibility. The AKSLD-2000 dataset, adopted from ref. [36], is included as a representative example of a low-resource dataset construction process. Although the original work in ref. [36] introduced this dataset, it did not provide a detailed description of its structure, collection methodology, or linguistic validation procedure; the dataset repository is publicly available at https://huggingface.co/datasets/Bakzhan/AKSLD-2000 (accessed on 23 May 2026). To address this gap, the present review provides a comprehensive characterization of AKSLD-2000 alongside comparable corpora, enabling a structured cross-dataset comparison.

As shown in Table 3, most publicly available sign language datasets focus on resource-rich languages such as American Sign Language (ASL) and Turkish Sign Language, with large numbers of samples and a diverse range of sign language speakers. In contrast, the two available datasets—KSL [62] and AKSLD-2000 [36]—are considerably more limited in scale. The KSL dataset provides a baseline for 30 sign classes with annotations that are independent of native sign language speakers, making it suitable for word-level classification tasks. However, its limited vocabulary and lack of sentence-level annotations significantly limit its applicability to current research in the field of SLR. The AKSLD-2000 dataset addresses part of this gap by offering 2000 linguistically validated units with 3D avatar-based visualization, supporting sentence-level recognition. MS-ASL, WLASL, AUTSL, LSA64, and ArSL2018 datasets have been widely adopted as benchmark datasets in recent sign language recognition studies. Nevertheless, both datasets remain insufficient in terms of signer diversity, recording conditions, and overall corpus size relative to high-resource benchmarks such as WLASL (21,083 videos, 119 signers) or AUTSL (38,336 samples, 43 signers). This gap highlights the urgent need to create a large, multimodal and linguistically sound corpus for Kazakh and other less resource-intensive sign languages, which is cited as a major research gap in this review.

To illustrate the structure of AKSLD-2000 concretely rather than only in aggregate form, a representative sample record from the corpus consists of the following fields: a gloss label (the Kazakh word or phrase denoted by the sign), a unique video identifier, a signer identifier, clip duration (typically 2–4 s), frame rate and resolution, a word-level annotation tag, and a corresponding 3D avatar-rendered animation sequence generated from the same keypoint trajectories as the RGB recording. This dual RGB-plus-avatar structure is what allows AKSLD-2000 to support both discriminative recognition tasks and generative avatar-based translation, unlike single-modality datasets such as WLASL or AUTSL. In terms of volume, AKSLD-2000 (2000 samples, 2000 classes) is approximately 2.5× larger than the existing KSL dataset [62] (~800 samples, 30 classes) but remains 10.5× smaller than WLASL (21,083 samples), 19.2× smaller than AUTSL (38,336 samples), and 12.8× smaller than MS-ASL (25,513 samples), underscoring the scale gap that still separates Kazakh Sign Language resources from high-resource benchmark corpora.

## 7. Kazakh Sign Language: Comparative Analysis and Research Trends (2021–2026)

KSL remains one of the least explored sign languages in the field of automatic sign language recognition. Unlike high-resource sign languages such as American Sign Language (ASL) and Turkish Sign Language (represented in this review by the AUTSL dataset), KSL suffers from limited publicly available datasets, a small number of dedicated benchmark studies, and the absence of standardized evaluation protocols. Consequently, the deep learning advances that have driven progress for high-resource sign languages have been adopted more slowly, and only recently, for KSL.

Table 4 contrasts the general research profile of high-resource sign language recognition—as represented by the ASL, WLASL, and AUTSL datasets discussed in Section 6—with the current state of KSL research identified in this review.

These differences indicate that the principal limitation of KSL research is no longer the recognition architecture itself, but the availability of sufficiently large, diverse, and standardized datasets. Modern transformer-based and multimodal models have demonstrated strong performance on large-scale benchmarks, but their effectiveness depends heavily on extensive annotated training data—a requirement that remains difficult to satisfy for KSL, since collecting, annotating, and linguistically validating sign language corpora is both time-consuming and resource-intensive.

Beyond this general comparison, it is informative to situate KSL-specific studies against other low- and medium-resource sign languages for which dedicated recognition studies appeared within the same five-year review window (2021–2026), since the high-resource benchmarks above (WLASL, AUTSL, MS-ASL) were themselves established earlier (2018–2020) and are not directly comparable in terms of research maturity. Table 5 presents this comparison.

This comparison indicates that KSL entered the field of dedicated, sign-language-specific recognition research roughly one to two years after comparably under-resourced languages such as Korean and Pakistani Sign Language, and that its trajectory has so far combined dataset creation (AKSLD-2000) with recognition modeling, whereas the Korean and Pakistani studies focused solely on recognition accuracy without a comparable public dataset contribution. This suggests that KSL research, while later-starting, is following a more dataset-conscious development path than some of its peer low-resource languages—a distinction directly relevant to the dataset-scarcity gap identified throughout this review.

Taken together, the two comparisons in this section show that the principal bottleneck for KSL is not modeling capacity but data availability and standardization. Future KSL research should therefore prioritize: (1) larger, signer-independent benchmark datasets; (2) standardized evaluation protocols that enable direct comparison with international benchmarks; (3) multimodal data acquisition combining RGB, depth, and wearable sensing; and (4) adoption of the architectural paradigms that have driven recent progress in high-resource sign languages, including transformer-based and graph-based models, synthetic data augmentation, and explainable AI techniques.

Several additional dimensions merit discussion beyond dataset volume. Regarding native signer participation, data collection for the KSL corpus referenced in this review was conducted with written informed consent from all participating signers, reflecting common practice for engineering-oriented corpus collection rather than clinical research; broader community involvement remains an opportunity for future KSL data collection efforts. Regarding linguistic validation, future corpus expansion would benefit from involving certified KSL interpreters or linguists in the annotation review process to confirm that recorded signs correspond to standard KSL usage rather than idiosyncratic or regionally specific variants, since—as with many under-documented sign languages—dialectal variation across different regions of Kazakhstan has not yet been systematically studied and may affect cross-corpus generalization. Regarding privacy, video and motion-capture recordings of signers constitute biometric data; safeguards should include anonymized storage identifiers (decoupling signer identity from raw video), participant control over data retention, and restricted-access distribution agreements for any released corpus, rather than fully open public release by default. Regarding transfer learning opportunities, the pose-based and keypoint-based feature representations used in several KSL studies (Table 5) are architecturally compatible with models pretrained on higher-resource sign languages (e.g., ASL via WLASL), suggesting that cross-lingual transfer learning—fine-tuning a model pretrained on a large-vocabulary sign language corpus using a small KSL-specific dataset—is a promising direction for mitigating the data scarcity documented throughout this review, rather than training KSL models from scratch on limited data.

Building on the comparative analysis above, three concrete directions can advance low-resource SLR dataset development beyond general calls for “more data.” First, data collection should follow a community-engaged protocol involving native signers from the outset of corpus design, with documented informed consent covering not only the recording itself but also potential future re-use of the collected video and motion data for downstream tasks such as avatar-based generation, following the informed-consent practice used during data collection for this study. Second, annotation should adopt a standardized schema recording manual parameters (handshape, location, movement) separately from non-manual markers (facial expression, mouth patterns, posture), using established sign-language annotation tools such as ELAN with ID-gloss conventions, rather than word-level translation labels alone. Third, benchmarking should adopt signer-independent train/validation/test splits, following the practice established by WLASL and AUTSL, report accuracy alongside vocabulary size and signer count as contextual metadata (Table 2), and, where feasible, fix a shared evaluation protocol so that successive KSL studies can be compared on equal terms rather than each introducing an ad hoc split, as is currently the case across the three KSL-specific studies in Table 5.

## 8. Discussion

A key requirement for developing an effective SLR system based on sensor technologies is the availability of representative and diverse datasets [107]. In sensor systems, this includes inertial signals, motor skill signals, and other cues; in the case of multimodal approaches, this includes synchronized visual data reflecting both passive and non-passive gesture elements. The lack of complete datasets containing annotated dynamic sequences significantly limits the model’s ability to identify complex patterns and generalize its results to new conditions.

Another challenge is the need to comprehensively account for the spatiotemporal structure of gestures. Sensor systems often record individual aspects of movement (e.g., trajectory and muscle activity), but if they are not integrated with other modalities, important information about facial expression, hand orientation, and contextual features of gesture execution can be lost. This underscores the importance of developing multimodal solutions capable of integrating various types of data.

The diversity of users and signal recording conditions is also a critical factor. Sensory data is sensitive to device positioning, individual motor skills, and gesture styles, reducing the robustness and generalization capabilities of the device model. Furthermore, the limited computing resources of wearable devices place additional demands on algorithm performance and real-time performance. Sign language remains an important means of communication for people with hearing and speech impairments, demonstrating the high societal significance of this technology. Despite recent advances, sensory-based gesture recognition technology still faces a number of unresolved challenges, including insufficient data standardization, limited scalability, and poor integration of different modalities. This highlights the need for further research to develop adaptive, robust, and interpretable systems capable of performing effectively in real-world settings.

A prerequisite for creating effective sensory-based gesture recognition systems is the availability of representative and sufficiently diverse datasets. In sensory approaches, this requirement is not limited to inertial signals, myoelectric, and other physiological signal modalities; For multimodal systems, it also includes synchronized visual data capturing both passive and non-passive movements. The lack of complete, annotated datasets containing dynamic gesture sequences significantly limits the model’s ability to identify complex behavioral patterns and generalize them to unknown situations. Another challenge is comprehensively modeling the spatial and temporal structure of gestures. Sensor-based systems often capture individual aspects of movement, such as trajectories and muscle activity; however, without integrating additional modes, important information about facial expression, hand orientation, and situational variables of performance can be lost. This highlights the importance of developing multimodal solutions capable of systematically combining heterogeneous data streams.

A systematic analysis of existing sensor and multimodal SLR methods has revealed deep-rooted scientific and practical limitations. First, most of the reviewed methods rely on static or simplified modal fusion strategies, such as feature linking and fixed-weight aggregation, and do not consider the context-dependent contributions of individual perceptual channels during gesture execution [84,88,94,101].

Second, most of the systems studied lack time-dependent modeling or implement it incorrectly, which limits their ability to correctly handle complex continuous gestures and extended sign language contexts [38,68,81,85]. Even architectures incorporating recursive components exhibit limitations in capturing long-term dependencies and hierarchical linguistic structures of continuous sign language [88,101].

Third, many methods rely on hand-crafted feature engineering and shallow classification models, which limits their ability to extract complex nonlinear and hierarchical behavioral representations [71,84,85]. This directly degrades the generalization ability of models across user diversity and data collection methods [29,67]. Furthermore, scalability and computational efficiency remain unresolved issues, particularly in systems working with large gesture vocabularies and systems using template matching and kernel-based classifiers [67,68]. In addition to the limited availability of annotated training data, sensor signals are inherently sensitive to configuration changes, calibration drift, and user-specific biomechanical differences, further hindering practical application [29,85].

Finally, most approaches inadequately model cross-modal dependencies and biomechanical correlations between heterogeneous signal types and largely ignore non-passive elements of singing, such as facial expressions and posture, thereby reducing the integrity of the resulting representations [59,67].

These limitations collectively point to the need for adaptive multimodal fusion mechanisms, time-aware deep learning architectures integrating attention mechanisms and transformer-based encoders, and domain-adaptive strategies that enable robust cross-user generalization and efficient real-time performance [63,77].

### Emerging AI Directions for Future Sign Language Recognition Systems

Recent advances indicate that future sign language recognition (SLR) systems will increasingly integrate Explainable Artificial Intelligence (XAI), Generative Artificial Intelligence (GenAI), and Responsible Artificial Intelligence (RAI). Although recent research has primarily focused on improving recognition accuracy through deep learning and multimodal architectures, emerging studies suggest that future SLR systems should also emphasize model transparency, trustworthy decision-making, synthetic data generation, fairness, and ethical deployment. Explainable AI is gradually becoming an important component of modern SLR systems. Recent studies have demonstrated that explainability techniques such as class activation maps (CAM), Grad-CAM, and interpretable prediction modules can identify the visual regions and temporal segments contributing most to recognition decisions, thereby improving model transparency and user confidence [108,109]. While the current body of research remains relatively limited, these approaches represent an important transition from purely accuracy-oriented evaluation toward interpretable and trustworthy recognition models. Generative AI has emerged as one of the most promising approaches for addressing the scarcity of annotated sign language datasets. Recent research demonstrates that synthetic corpus generation, adversarial data augmentation, diffusion-based sign production, and motion generation can substantially improve training diversity while reducing the need for expensive manual annotation [110,111,112,113]. These approaches are particularly valuable for low-resource sign languages such as KSL, where collecting large-scale multimodal datasets remains both costly and time-consuming. Consequently, synthetic data generation represents a promising strategy for improving model robustness, reducing overfitting, and facilitating the development of standardized benchmark datasets. Responsible AI represents another emerging research direction that has received comparatively little attention within the SLR community. Beyond maximizing recognition accuracy, future SLR systems should also address fairness across different signers, demographic diversity, privacy preservation during data collection, transparent dataset documentation, and reproducibility of experimental results [114,115]. Incorporating these principles will improve the reliability, trustworthiness, and real-world applicability of sensor-based and multimodal SLR systems, particularly for assistive technologies intended for everyday communication. Overall, Explainable AI, Generative AI, and Responsible AI collectively represent promising research directions that extend current SLR research beyond recognition performance toward transparent, scalable, trustworthy, and socially responsible intelligent communication systems.

## 9. Conclusions

This review synthesized 76 sensor-based and multimodal sign language recognition studies published between 2021 and 2026, organized into four taxonomy categories (wearable sensor-based, contactless non-visual, vision-based, and multimodal systems) and three methodological categories. Among the 76 systems catalogued by the methodological approach in Appendix B, deep learning methods dominate the field (61 systems, 80%), while machine learning (8 systems, 11%) and conventional techniques (7 systems, 9%) remain a small but persistent minority, typically favored for their lower computational load in resource-constrained wearable deployments. Reported accuracy across the reviewed systems ranged from as low as 77% under event-based or cross-domain evaluation conditions to over 99% under controlled, single-signer conditions (Table 2), a spread that reflects differences in vocabulary size, signer independence, and evaluation protocol rather than a straightforward ranking of methods. Latency and computational cost, reported systematically in Appendix B, further show a clear trade-off: the majority of systems achieve real-time operation at low-to-medium computational load, while the highest-accuracy transformer- and GAN-based architectures typically incur high-to-very-high computational cost, a trade-off directly relevant to real-world deployability that is rarely discussed explicitly in the primary literature itself. Across this literature, three limitations recur regardless of sensing modality: static or fixed-weight multimodal fusion strategies that do not adapt to context, limited temporal modeling that fails to distinguish static from continuous signing, and poor cross-user generalization due to unmodeled sensor-placement and biomechanical variability.

The case of Kazakh Sign Language, examined in dedicated comparative analysis (Section 7), illustrates these gaps concretely: only three KSL-specific studies were identified within the entire five-year review window, and the resulting AKSLD-2000 corpus, while roughly 2.5 times larger than the previously existing KSL dataset, remains an order of magnitude smaller than high-resource benchmarks such as WLASL and AUTSL (Table 3). This mirrors the broader pattern identified across the review: dataset scarcity and lack of standardization, not modeling capacity, are the primary bottleneck constraining sign language recognition for the majority of the world’s sign languages. Future research should therefore prioritize community-engaged, signer-diverse dataset collection with standardized annotation and benchmarking protocols, alongside continued architectural progress in adaptive multimodal fusion, temporal modeling for continuous signing, and cross-lingual transfer learning from high-resource to low-resource sign languages.

## Figures and Tables

**Table 1:** Comparison of the present review with closely related survey articles.

Dimension	Amin et al. [4]	Najib et al. [7]	This Review
Publication year	2022	2025	2026
Papers covered	~50	~70	76
Period covered	Up to 2022	Up to 2024	2021–2026
*Scope and taxonomy*			
Structured taxonomy of SLR systems	Not applicable	Not applicable	Yes
Focus on wearable sensor-based systems	Partial	Not applicable	Exclusive
Focus on contactless non-visual sensing systems	Not applicable	Not applicable	Exclusive
Focus on vision-based systems	Partial	Partial	Exclusive
Focus on multimodal systems	Not applicable	Not applicable	Exclusive
*Methodology*			
Systematic PRISMA methodology	Not applicable	Not applicable	Yes
Methodological approach classification	Not applicable	Partial	Yes, three-category
*Technical coverage*			
Comparative analysis based on conventional techniques	Partial	Partial	Yes, comprehensive
Comparative analysis based on machine learning	Partial	Yes	Yes, comprehensive
Comparative analysis based on deep learning	Not applicable	Partial	Yes, comprehensive
*Additional dimensions*			
Low-resource sign languages	Not addressed	Not addressed	Yes, dedicated focus
Qualitative discussion of potential biases	Not addressed	Not addressed	Yes
Integrative discussion with roadmap	Not applicable	Not applicable	Yes

**Table 2:** Reported accuracy across representative sensor-based, contactless, and vision-based SLR systems reviewed in Section 3.

Reference	Methodology	Modality	Reported Accuracy	Task/Context
[27]	ML	Wearable (magnetic positioning)	~97%	Static alphabet
[29]	DL (ANN)	Wearable (smart glove)	98.5%	Static alphabet (26 letters)
[32]	ML (RF/SVM/kNN)	Wearable (Flex sensors)	99%	Dynamic ASL words
[40]	ML (sensor fusion)	Wearable (tactile textile)	94.66%	Gesture recognition
[42]	DL (attention-based)	Contactless (RFID)	99.17%/97.5%/96.67%	Overall/new environments/new users
[43]	DL (CNN-BiLSTM)	Contactless (RF)	96.8%/96.3%	Overall/new users
[44]	DL (CRNN-CTC)	Vision	98.8%/92.4%/86.3%	Isolated/continuous/complex sequences
[45]	DL (SNN)	Vision (event-based/DVS)	77%	Event-based gesture recognition
[46]	Conventional (DTW)	Vision (Leap Motion)	95.17%	Large-vocabulary gestures
[59]	DL (STGCN-LSTM)	Vision (skeleton)	98.42%	Skeleton-based recognition
[68]	DL (Transformer/GRU)	Vision	99.87%/100%	Korean Sign Language
[77]	DL (SLATN, Transformer)	Vision	82.66%	Pakistani SL (PkSLMNM), manual + non-manual
[82]	DL (I3D + LSTM)	Multimodal (RGB + pose)	89.3%	Multimodal fusion
[93]	DL (CNN-LSTM)	Wearable (tensor + IMU)	>99%	ASL letters/words
[99]	ML (HMM)	Wearable (sEMG + accelerometer)	90.41%	Gesture recognition

**Table 3:** Comparative analysis of selected sign language datasets mentioned in this review.

Reference	Dataset	Language	Classes	Samples	Modality	Annotation	Signers	Access
[9]	GSL	Greek	310	40,785	RGBD + skel.	Sentence-level	7	Public
[36]	AKSLD-2000	Kazakh	2000	2000	RGB + 3D avatar	Word-level	Mult.	Public
[62]	KSL	Kazakh	30	~800	Video + skeleton	Word-level	Mult.	Public
[102]	MS-ASL	American	1000	25,513	RGB	Word-level	222	Public
[103]	WLASL	American	2000	21,083	RGB	Word-level	119	Public
[104]	AUTSL	Turkish	226	38,336	RGB-D	Word-level	43	Public
[105]	LSA64	Argentinian	64	3200	RGB	Word-level	10	Public
[106]	ArSL2018	Arabic	32	54,049	RGB	Letter-level	40	Public

**Table 4:** Qualitative comparison of high-resource sign language recognition research and current Kazakh Sign Language research.

Aspect	High-Resource SLR (ASL, WLASL, AUTSL)	Kazakh Sign Language
Dataset availability	Large public benchmark datasets (21,000+–38,000+ samples)	Limited public datasets (≤ 2000 samples)
Vocabulary size	Hundreds to thousands of signs	30–2000 signs, mostly word-level
Number of signers	Dozens to hundreds (43–222 signers in datasets reviewed)	Small, non-disclosed signer pools
Recognition task	Isolated and continuous/sentence-level SLR	Primarily isolated, word-level signs
Sensing modalities	RGB, depth, skeleton, multimodal fusion	Mainly RGB and pose/keypoint-based
Modern architectures	CNNs, Transformers, GCNs, multimodal fusion	MediaPipe keypoints, BiLSTM, YOLO-Pose
Benchmark protocols	Standardized public benchmarks with signer-independent splits	No widely adopted benchmark or standardized split

**Table 5:** Comparative timeline of low- and medium-resource sign language recognition studies published within the 2021–2026 review window.

Sign Language	Reference	Year	Method	Reported Accuracy	Contribution Type
Korean	[68]	2023	Pose estimation + attention network	99.87%/100%	Recognition method
Pakistani	[77]	2023	SLATN (action transformer)	82.66%	Recognition method
Kazakh	[62]	2024	MediaPipe keypoint pipeline	Not reported as a single figure	Recognition method
Kazakh	[36]	2025	AKSLD-2000 corpus	-	Dataset resource
Kazakh	[79]	2025	YOLO-Pose + BiLSTM + optical flow	Not reported as a single figure	Recognition method

## Data Availability

The source dataset is publicly available at: https://huggingface.co/datasets/Bakzhan/AKSLD-2000 (accessed on 23 May 2026).

## Appendix A

The source figures used in this review are presented below.

## Appendix B

The complete source table is presented below.

**Table A1:** Comparative Analysis of Sign Language Recognition Systems Based on Conventional Techniques.

Ref	Type of Method	Sign Language Units	Model	Algorithm	Architecture	Modality	Technique	Latency	Computational Load	Strengths	Limitations
[26]	Conventional	31 letters	SVM-based gesture classifier	Support Vector Machine (SVM)	Feature extraction + SVM classification	Single accelerometer	Signal-based classification with statistical features	Real-time	Low	Extremely simple, single sensor	Limited gesture complexity
[34]	Conventional	54 different alphabetic gestures (28 gestures for ArSL and 26 gestures for ASL)	Rule-based system	Rule-based + threshold	IoT system	Flex + IMU	Signal mapping + TTS	Real-time	Low	Mobile + bilingual	Static gestures
[41]	Conventional	26 letters	Rule-based	Rule-based threshold	Rule-based	Flex + IMU	Threshold-based signal mapping	Real-time	Very low	Simple	No generalization
[46]	Conventional	176 words	DTW-based recognition system	Dynamic Time Warping (DTW)	Preprocessing + DTW alignment pipeline	3D hand skeleton (Leap Motion)	Temporal sequence alignment	Real-time	Low	High accuracy, invariant to speed, no lighting dependency	Sensor noise, occlusion, temporal variability
[83]	Conventional	82 signs	Classical hybrid	MSL medical DTW-kNN	kNN + DTW fusion	Leap Motion + Kinect	Classical ML	Real-time	Low	Medical application robust	Small vocabulary
[84]	Conventional	20 gestures	DTW-based system	Weighted DTW	Non-deep learning temporal alignment	Flex + IMU sensors	Sensor fusion + weighted similarity	Real-time	Low	Noise robustness, low cost, illumination-independent	Small dataset, limited gestures
[92]	Conventional	13 gestures	Classical pattern matching	Pattern matching	Template-based	Flex + IMU	Template-based gesture matching	Real-time	Low	Simple, low cost	Small vocabulary

**Table A2:** Comparative Analysis of Sign Language Recognition Systems Based on Machine Learning.

Ref	Type of Method	Sign Language Units	Model	Algorithm	Architecture	Modality	Technique	Latency	Computational Load	Strengths	Limitations
[27]	Machine Learning	24 alphabet letters	SVM + k-NN + localization	SVM, k-NN + LM	Classical ML	Magnetic sensors	Position estimation	Real-time	Medium	Robust occlusion	Magnetic noise
[32]	Machine Learning	23 gestures	Random Forest/SVM-based classification system	Random Forest, SVM	Classical ML pipeline	Flex sensors	Temporal signal classification	Real-time	Low	High accuracy, minimal sensors	No spatial info
[37]	Machine Learning	27 gestures	Random Forest (primary), SVM (baseline)	Random Forest, SVM	Classical ML pipeline	Flex + IMU glove	Feature selection + classification	Real-time	Low	High accuracy, low cost	Missing finger sensors
[38]	Machine Learning	20 gestures	SVM, KNN, LDA, Decision Tree and Ensemble-based	SVM, KNN, LDA, Ensemble	Classical ML pipeline	Flex + IMU glove	Sensor fusion + feature extraction	Real-time	Low	High accuracy, real-time, robust	Limited scalability
[85]	Machine Learning	80 words	Ensemble ML classifiers (RF, SVM, k-NN, Naive Bayes)	k-NN, RF, SVM	Signal processing + ML classification pipeline	EMG + IMU wearable	Temporal feature extraction	Real-time	Low	Very fast, scalable gestures	Device dependency
[86]	Machine Learning	26 gestures	SVM, KNN and FNN-based	SVM, KNN, FNN	Classical ML pipeline	Flex + IMU glove	Sensor fusion + filtering + feature engineering	Real-time	Low	Robust to noise, drift compensation	Sensor sensitivity
[87]	Machine Learning	10 sign words	MLP	MLPFFNN	Feedforward NN	Flex + IMU	PWM normalization + classification	Real-time	Low	Low-cost wearable system	Static gestures only
[88]	Machine Learning	10 sign words	Hybrid ML pipeline	RF, SVM, k-NN, MLP	Hybrid ML pipeline	Flex + IMU glove	Sliding window + fusion	Real-time	Low	Works for ASD gestures	Similar gesture confusion

**Table A3:** Comparative Analysis of Sign Language Recognition Systems Based on Deep Learning.

Ref	Type of Method	Sign Language Units	Model	Algorithm	Architecture	Modality	Technique	Latency	Computational Load	Strengths	Limitations
[28]	Deep Learning	50 words and 20 sentences	CNN	CNN	Deep model	TENG + IMU	Segmented + continuous	Real-time	Medium	VR integration	Segmentation complexity
[29]	Deep Learning	26 letters	Artificial Neural Network	ANN	Neural network	Yarn-based strain sensors	Resistive signal mapping	Real-time	Low	Fast, flexible, scalable sensors	Manufacturing complexity
[30]	Deep Learning	50 sentences	BLSTM	BLSTM + CTC	Sequence model	Smartwatch IMU	Sequence learning	Real-time	Low	Portable	Misses finger motion
[31]	Deep Learning	26 letters	MLP	MLP (SCG)	Feedforward NN	Flex + IMU	Feature-based static classification	Real-time	Low	Simple, low-cost	Only static gestures
[33]	Deep Learning	10 gestures	IRDC-Net (CNN-based)	CNN (Inception + dilation)	Deep CNN	sEMG 8-channel	Feature extraction	Real-time	Medium	High accuracy	User dependency
[35]	Deep Learning	300 commonly used sentences	CNN–BiLSTM–CTC	CNN-BiLSTM-CTC	Encoder model	IMU wearable	Sequence alignment	Near real-time	High	Sentence-level recognition	High computation
[36]	Deep Learning	Sentence-level	Transformer	Attention Mechanism	Encoder–Decoder Transformer	Text + Gloss	Parallel Corpus Learning	Medium	High	High translation accuracy; specialized KSL parser; large-scale parallel corpus; supports communication accessibility	Limited annotated data
[39]	Deep Learning	11 common words	Embedded MLP	MLP	Embedded NN	Hall sensors + IMU	Magnetic classification	Real-time	Low	Low cost	Magnetic interference
[40]	Deep Learning	24 letters (excluding j and z), 3000 samples	CNN-SVM	CNN + SVM	Hybrid model	Hydrogel tactile	Pressure classification	Real-time	Medium	Tactile robustness	Moisture sensitivity
[42]	Deep Learning	6 signs	Convolutional deep learning model with attention	RF-CSign	Convolutional deep learning + attention (large-kernel CNN + NAM)	RFID signals	Domain adaptation/transfer learning	Not specified	Medium-High	High accuracy, cross-domain robustness	Needs data for each environment
[43]	Deep Learning	6 sign phrases	CNN-BiLSTM	RF-SL	CNN-BiLSTM + Attention	RFID signals	RF signal processing + CNN-BiLSTM	Not specified	Medium	Privacy-preserving, robust to lighting	Sensitive to fine finger motion, heavy compute
[44]	Deep Learning	15 single gestures	Ultrasonic CRNN–CTC model with GAN-based	UltrasonicGS	CRNN + CTC + GAN augmentation	Ultrasonic signals	Sequence modeling + GAN data augmentation	Not specified	Medium	Contactless, low-cost, good continuous recognition	Noise, alignment complexity
[45]	Deep Learning	64 commonly used words	Spiking Neural Network	DVS-SNN SLR	Spiking Neural Network (STBP)	Event camera (DVS)	Spatio-temporal spike learning	Low	Low	Low power, works in low light	Noise sensitivity, training complexity
[47]	Deep Learning	26 letters and 10 digits	Self-attention CNN–LSTM architecture with hybrid optimization	CNNSa-LSTM	CNN + Self-Attention + LSTM	RGB video + optical flow	Hybrid deep learning + optimizer	Not specified	High	Very high accuracy, robust	High computation cost
[48]	Deep Learning	26 letters	CNN–ViT–BiGRU with metaheuristic optimization	ISLRHP-HMOADL	ResNeXt + VGG19 + ViT + BiGRU	RGB images	Metaheuristic optimized deep learning	Not specified	Very High	Very high accuracy, strong feature fusion	Complex, heavy model
[49]	Deep Learning	KArSL dataset 190 signs that comprise 30 digit 537 signs, 39 letter signs, and 121 word signs and second KArSLdataset-502 signs, LSA64 dataset-64 signs	Two-stream CNN	DMN-AMN-SRN	Multi-stream CNN-based hierarchical network	RGB video	Key pose learning + sequence modeling	Not specified	Medium	Works with few frames, efficient	Less effective on complex gestures
[50]	Deep Learning	50 signs	CNN (InceptionV3)	InceptionV3 ArSL	InceptionV3 CNN (Transfer Learning)	RGB images	Classification	Low	Medium	Simple, strong generalization	Background sensitivity
[51]	Deep Learning	100 words	Attention-enhanced CNN–LSTM architecture	MobileNetV2 CNN-LSTM	CNN + LSTM + Attention	RGB video	Spatio-temporal deep learning	Medium	Low-Medium	Efficient, lightweight	Moderate accuracy
[52]	Deep Learning	BosphorusSign22k consisting of 744 signs, LSA64 dataset-64 signs, INCLUDE50 dataset- 263 signs	EfficientNet and BiLSTM	EfficientNet-BiLSTM	EfficientNet + BiLSTM + Attention	RGB video	Deep feature extraction + temporal modeling	Medium	Medium	Accurate, parameter-efficient	Lighting sensitivity
[53]	Deep Learning	44 words and 6 digits	LSTM	MediaPipe CNN-LSTM	CNN + LSTM + Attention	Keypoints (MediaPipe)	Pose-based sequence learning	Low	Low	Real-time, robust	Limited vocabulary
[54]	Deep Learning	11 words, 1100 samples	combined LSTM and GRU	DeepSign	CNN (Inception/ResNet) + LSTM	RGB video	Transfer learning + sequence model	Medium	High	Real-time, robust	Computational cost
[55]	Deep Learning	26 letters	CNN	Basic CNN SLR	CNN	RGB images	Classification	Low	Low	Simple, fast	No dynamic gestures
[56]	Deep Learning	26 letters	Hybrid deep learning model combining LSTM and YOLOv6	Hybrid ASL SLR	LSTM + YOLOv6	RGB video + skeleton	Detection + sequence modeling	Real-time	Medium	Real-time, hybrid robustness	Complex pipeline
[57]	Deep Learning	13 sign gestures	Modified GRU (MOPGRU)	MOPGRU	Modified GRU	Skeleton (MediaPipe)	Sequence modeling	Real-time	Low	Fast convergence, high accuracy	Small dataset
[58]	Deep Learning	150 signs	RNN	MSL RNN system	RNN	Synthetic video sequences	Sequence learning	Not specified	Medium	Dataset-scale learning	Hard generalization
[59]	Deep Learning	500 sign words	Spatial–Temporal Graph Convolutional Network (STGCN)—LSTM	Spatial–Temporal Graph Convolution + LSTM	Spatio-temporal graph neural network with recurrent (LSTM) fusion	Skeleton data	Graph-based spatio-temporal learning + temporal sequence modeling	Medium	Medium	High accuracy, structural modeling	Computational complexity
[60]	Deep Learning	10 targeted words	Hybrid CNN and LSTM	ArSL hybrid CNN-LSTM	CNN + LSTM	RGB images/video	Hybrid deep learning	Not specified	Medium	Good static + dynamic handling	Limited dataset
[61]	Deep Learning	PHOENIX14 and PHOENIX14-T 6841/8247 sentences, CSL-Daily dataset-20,654 sentences, CSL dataset-100 sentences	Self-Enhancing Network (SEN)	Lightweight spatial–temporal feature extraction + attention-based refinement + temporal self-emphasis	Attention-based spatio-temporal CNN with temporal self-enhancement module	RGB video	Spatial + temporal attention mechanisms	Medium	Low–Medium	Efficient, no pose dependency, adaptive attention, scalable to large datasets	Sensitive to video quality, high data demand, less interpretable
[62]	Deep Learning	30 gestures	LSTM	KSL LSTM system	LSTM	Skeleton (MediaPipe)	Temporal sequence classification	Real-time	Low	Simple real-time system	Limited gestures
[63]	Deep Learning	WLASL-100, WLASL-300, WLASL-1000, and WLASL2000, where 100, 300, 1000, and 2000 signs, LSA64 dataset-64 signs	GCN-based framework	Graph Convolutional Network + Attention	Multi-stream spatio-temporal GCN with attention fusion	Skeleton joints	Pose-based graph learning with attention	Low	Low	Fast, robust to background	Depends on pose quality
[64]	Deep Learning	WLASL-2000 signs, MSL-30 signs, ASLLVD-2745 signs, and PSL-19 signs.	Two-stream Multi-stage GCAR (Graph Convolutional Attention Residual model)	GCAR model	Multi-stage GCN + Attention	Skeleton	Two-stream graph learning	Real-time	High	Very robust, full-body modeling	Heavy computation
[65]	Deep Learning	293 gestures	SA-3DGCN (Spatial Attention 3D GCN)	3D Graph Convolutional Network + CNN + Attention	Hybrid GCN + 3D-CNN + Spatial Attention network	Skeleton 3D	Attention-enhanced spatio-temporal graph learning	Medium	Medium	Accurate, invariant to viewpoint	Noise in keypoints
[66]	Deep Learning	KArSL dataset containing 100 words, LSA64 dataset-64 signs	Transformer	Pose Transformer SLR	Transformer + LSTM + TCN	Pose keypoints	Sequence modeling	Real-time	Low-Medium	Very high accuracy	Needs good keypoints
[67]	Deep Learning	RWTH-PHOENIX-Weather 2014T dataset-40k videos for sentence level, ISL-CSLTR dataset-100 sentences, How2Sign dataset 2456 videos for sentences.	CNN–BiLSTM (recognition)-NMT–GAN (generation)	CNN-STGCN Transformer GAN	CNN + STGCN + Transformer + GAN	RGB video + skeleton	Multi-task learning (SLR + SLT + SLG)	High	Very High	End-to-end system	Very complex
[68]	Deep Learning	77 sign words	Two-stream GCN- Attention-based CNN	KSL GRU-Attention	GRU + Attention	Skeleton	Sequence learning	Real-time	Low	Efficient, accurate	Limited vocabulary
[69]	Deep Learning	6707 sign words	Swin Transformer-based Dual-View Spatio-Temporal WSLR Model	Dual-view Swin Transformer	Swin Transformer	Multi-view video	Transformer fusion	High	Very High	Multi-view robustness	Data heavy
[70]	Deep Learning	300 words	SNN	SNN SLR	Spiking Neural Network	RGB video	Energy-efficient temporal learning	Real-time	Low	Energy-efficient	Hard training
[71]	Deep Learning	RWTH-PHOENIX-Weather 2014 dataset-6841 sentence-level videos and a vocabulary of 1295 glosses, CSL dataset-100 predefined sentences, Signer Independent dataset-310 glosses.	SLRGAN (GAN-Bi-LSTM-Transformer)	SLRGAN	GAN + BiLSTM + Transformer	RGB video	Generative sequence learning	Not specified	Very High	Context-aware continuous SLR	Complex training
[72]	Deep Learning	BosphorusSign22k consisting of 744 signs, LSA64 dataset-64 signs, INCLUDE50 dataset- 263 signs	Dual-stream MambaVision-skeletal fusion model (AG-FiCL)	MambaVision AG-FiCL	Mamba SSM + fusion module	Multi-modal skeleton + video	State-space sequence modeling	Real-time	Medium	Efficient long sequence modeling	Novel approach risk
[73]	Deep Learning	KArSL-100 includes 100 signs, KArSL-190 expands to 190 signs, KArSL-502, the most extensive dataset, covers 30 digits, 40 letters, and 432 words.	LSTM	ArSL LSTM fusion	LSTM + feature fusion	RGB + keypoints	Feature-based classification	Real-time	Medium	Good multi-feature fusion	Low accuracy on large datasets
[74]	Deep Learning	40 words	Probabilistic Skeleton-based SLR Model	Probabilistic skeleton SLR	Statistical model + feature transform	Skeleton (Leap Motion)	Probabilistic time modeling	Real-time	Low	Robust feature modeling	Dataset-specific
[75]	Deep Learning	371 letters, 385 functional words, and 450 interrogative signs.	GmTC (Graph and Transformer-based CNN model)	MSL angular-BiLSTM	BiLSTM	Angular geometric descriptors	Compact feature sequence learning	Real-time	Very Low	Lightweight, high accuracy	Limited generalization
[76]	Deep Learning	500 words and 2000 sign language words	GCN	AMD-GCN	GCN + temporal conv + attention	3D hand skeleton	Graph temporal modeling	Medium	Medium	Accurate fine-grained gestures	Moderate compute
[77]	Deep Learning	7 gesture	Action Transformer Network (SLATN)	Transformer-based spatio-temporal attention + object localization head	Hybrid transformer architecture with dual attention streams	RGB video (hand + pose + facial features)	Spatio-temporal attention learning + multimodal action localization	Real-time	High	Captures both manual and non-manual gestures, high accuracy, attention-based interpretability	High computational cost, dataset dependency, complex training
[78]	Deep Learning	36 alphabetic and numeric signs, 12 gesture	Hybrid LSTM–3D CNN	LSTM + 3D CNN	Dual-branch hybrid architecture (static–dynamic split)	RGB video + skeleton (MediaPipe landmarks)	Hybrid spatio-temporal modeling	Real-time	Low	Edge deployment, low latency, balanced accuracy	Overfitting in LSTM, limited scalability
[79]	Deep Learning	42 alphabet and 7 common words	BiLSTM	KSL YOLO-BiLSTM	YOLOv8/11 Pose + BiLSTM	Skeleton + optical flow	Feature fusion temporal model	Real-time	Low	Real-time high accuracy	Occlusion sensitivity
[80]	Deep Learning	226 words and 2000 common words	3D ConvNet-Fusion	3D Deformable ConvNet Fusion Model	3D Conv + SSM + fusion	RGB + optical flow + skeleton	Multimodal spatio-temporal fusion with attention	High	High	Robust SOTA performance	Complex architecture
[81]	Deep Learning	500-word and 1100 unique signs	Hybrid CNN-GCN model	3D-CNN + GCN + Attention fusion	Multimodal deep fusion architecture	Video + skeleton	Multi-semantic feature fusion with attention	High	High	Very high accuracy	Heavy compute
[82]	Deep Learning	154 signs	3D-CNN-HMM	Multistream I3D-HMM	3D CNN + HMM + fusion	RGB + optical flow + skeleton	Multistream late fusion	High	High	Robust multi-stream	Complex pipeline
[89]	Deep Learning	digits (1–5) and 10 letters	MLP	MLPFFNN	Feedforward NN	Flex + IMU	PWM normalization	Real-time	Low	High accuracy	Limited dataset
[90]	Deep Learning	100 words and 50 sentences	LSTM	LSTM	Deep sequential model	Stretch sensors + microfluidic	Temporal sequence modeling	Real-time	Medium	Continuous SLR, signer adaptation	User dependency
[91]	Deep Learning	24 letters, words, phrases	LSTM	LSTM (quantized)	Embedded deep model	Conductive textile + IMU	Time-series modeling	Real-time	Low	On-device inference	Drift, limited memory
[93]	Deep Learning	18 gestures	CNN-LSTM	CNN + LSTM	Hybrid deep model	Flex + IMU	Spatio-temporal learning	Real-time	Medium	High accuracy	Limited generalization
[94]	Deep Learning	40 sentences	Transformer	Transformer	Encoder–decoder	IMU + sEMG	Self-attention	Real-time	High	Best translation	Data-hungry
[95]	Deep Learning	16 signs	LSTM-GRU ensemble	LSTM + BiLSTM + GRU	Ensemble RNN	Flex + IMU	CTC decoding	Real-time	Medium	Robust ensemble	Lower sentence accuracy
[96]	Deep Learning	41 words	CNN-LSTM	CNN + LSTM	Hybrid model	Flex + IMU	Spatio-temporal	Real-time	Medium	Dynamic gestures	User variability
[97]	Deep Learning	20 signs	CNN-BiLSTM-Attention	CNN-BiLSTM-Attention	Deep model	16 IMUs glove	Attention fusion	Real-time	High	Fine motion capture	Sensor-heavy
[98]	Deep Learning	10 gestures (words), 20,000 samples	BiLSTM	BiLSTM + CTC	Sequence model	IMU + sEMG	Multimodal fusion	Real-time	High	Continuous CSL	Speed sensitivity
[99]	Deep Learning	120 words	CNN-BiLSTM	CNN-BiLSTM + Attention	Multimodal fusion	EMG + IMU	Feature fusion	Real-time	High	Robust variation	Sensor shift
[100]	Deep Learning	26 letters	MLP	MLP	Feedforward NN	Potentiometer + FSR	Static classification	Real-time	Low	Low cost	No dynamic gestures
[101]	Deep Learning	32 gestures	CNN-BiLSTM	JSL CNN-BiLSTM multimodal	CNN + BiLSTM	RGB + wearable sensors	Multimodal fusion	Real-time	Medium	Handles occlusion well	Sensor dependency

## Appendix C. PRISMA 2020 Checklist

**Item**	**Checklist Item**	**Location in Manuscript**
Title		
1	Title	Identifies the report as a systematic review.
Abstract		
2	Abstract	Abstract; summarizes objectives, methods (PRISMA 2020, four databases, 76 studies), and main findings.
Introduction		
3	Rationale	Section 1; gap analysis vs. Amin et al. and Najib et al.
4	Objectives	Section 1; four contributions (taxonomy, methodological classification, KSL focus, research roadmap).
Methods		
5	Eligibility criteria	Section 2; inclusion criteria (i)–(iii) plus quality-assessment criteria.
6	Information sources	Section 2; Scopus, Web of Science, IEEE Xplore, Google Scholar.
7	Search strategy	Section 2; general Boolean search expression and description of database-specific syntax adaptation.
8	Selection process	Section 2; screening by all co-authors, record pool divided evenly, borderline cases resolved by discussion.
9	Data collection process	Section 2; full-text review against predefined criteria.
10a	Data items—outcomes	Section 2 defines the extracted outcome variables; Section 5, Table 2 and Appendix B report accuracy, latency, and computational cost where available.
10b	Data items—other variables	Appendix B; dataset, modality, architecture, signer independence.
11	Study risk of bias assessment	No formal study-level risk-of-bias assessment tool was applied. Methodological eligibility criteria are described in Section 2, and this distinction is explicitly acknowledged in the manuscript.
12	Effect measures	Not applicable—narrative/comparative synthesis, not meta-analysis.
13a	Synthesis—eligibility for synthesis	Section 5; comparative synthesis across taxonomy categories.
13b	Synthesis—data preparation	Appendix B; standardized comparison table.
13c	Synthesis—tabulation/visualization	Table 2 and Table 5, Appendix B.
13d	Synthesis—method used	Section 5; narrative synthesis with cross-method comparison.
13e	Synthesis—sensitivity analysis	Not applicable—narrative review, not meta-analysis.
14	Reporting bias assessment	No formal assessment of reporting bias was performed; this is acknowledged as a limitation of the review.
15	Certainty assessment	Not applicable—narrative review, not GRADE-based.
Results		
16a	Study selection—numbers	Figure 2; 665 identified → 98 duplicates removed → 567 screened → 398 excluded → 169 full-text assessed → 93 excluded → 76 included.
16b	Study selection—exclusion reasons	Figure 2, Section 2; title/abstract exclusions (publication type, language, non-sensor-based focus outside the predefined wearable sensor-based, contactless non-visual, vision-based, or multimodal SLR scope).
17	Study characteristics	Appendix B; full comparative table of 76 included studies.
18	Risk of bias in studies	No formal study-level risk-of-bias scores were generated. Potential dataset- and methodology-related biases are discussed qualitatively in Section 8 (Discussion).
19	Results of individual studies	Section 3; Appendix B.
20a	Results of syntheses	Section 5, Table 2.
20b	Results of syntheses—heterogeneity	Section 5; accuracy variability by vocabulary size, signer independence, evaluation protocol.
20c	Results of syntheses—sensitivity analyses	Not applicable.
20d	Results of syntheses—reporting biases	Not formally assessed; noted as limitation.
21	Certainty of evidence	Not applicable—narrative synthesis.
Discussion		
23a	Interpretation	Section 8 (Discussion).
23b	Limitations of included evidence	Section 8 (Discussion); limitations related to dataset scarcity, cross-user generalization, multimodal fusion, and evaluation heterogeneity.
23c	Limitations of review process	Section 2 and Section 8 (Discussion); limitations of the review process include the absence of a formal inter-rater agreement statistic and prospective protocol registration.
23d	Implications	Section Emerging AI Directions for Future Sign Language Recognition Systems and Section 9; future research directions and implications.
Other information		
24a	Registration	Not prospectively registered (e.g., PROSPERO); no formal protocol published prior to search.
24b	Protocol access	Not applicable—no protocol registered.
24c	Protocol amendments	Not applicable.
25	Support	Funding/Acknowledgments section.
26	Competing interests	Conflicts of Interest Statement.
27	Availability of data, code, materials	Appendix A and Appendix B; supplementary data embedded directly in manuscript.
